# Supplementary material for: RNA Polymerase II Phosphorylated on CTD Serine 5 Interacts with the Spliceosome during Co-transcriptional Splicing
Source: Mol Cell. 2018 Oct 18;72(2):369–379.e4. doi: 10.1016/j.molcel.2018.09.004 (PMC6201815; doi:10.1016/j.molcel.2018.09.004)
Supplement: Document S1. Figures S1–S4 [file mmc1.pdf]

**Molecular Cell, Volume *72***

## Supplemental Information

# RNA Polymerase II Phosphorylated on CTD Serine 5 Interacts with the Spliceosome during Co-transcriptional Splicing

**Takayuki Nojima, Kenny Rebelo, Tomás Gomes, Ana Rita Grosso, Nicholas J. Proudfoot, and Maria Carmo-Fonseca**

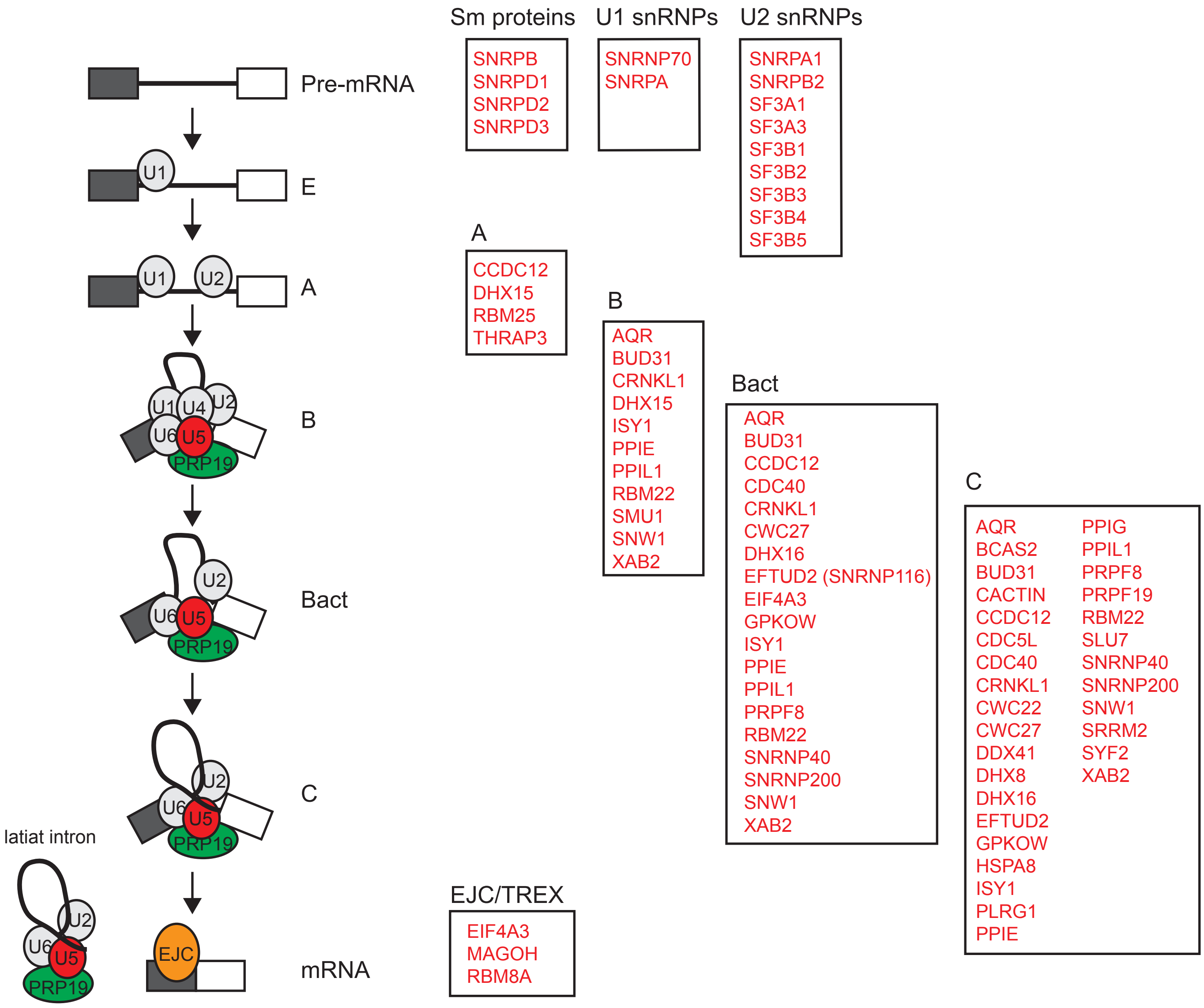


**Figure S1. Spliceosome proteins associate with S5P CTD Pol II (related to Figure 1).**
Schematic diagram of splicing steps with indicated U snRNAs. Peptides enriched by S5P CTD Pol II IP relative to mock IP include U snRNP proteins and components of spliceosome A, B, Bact, and C complexes.

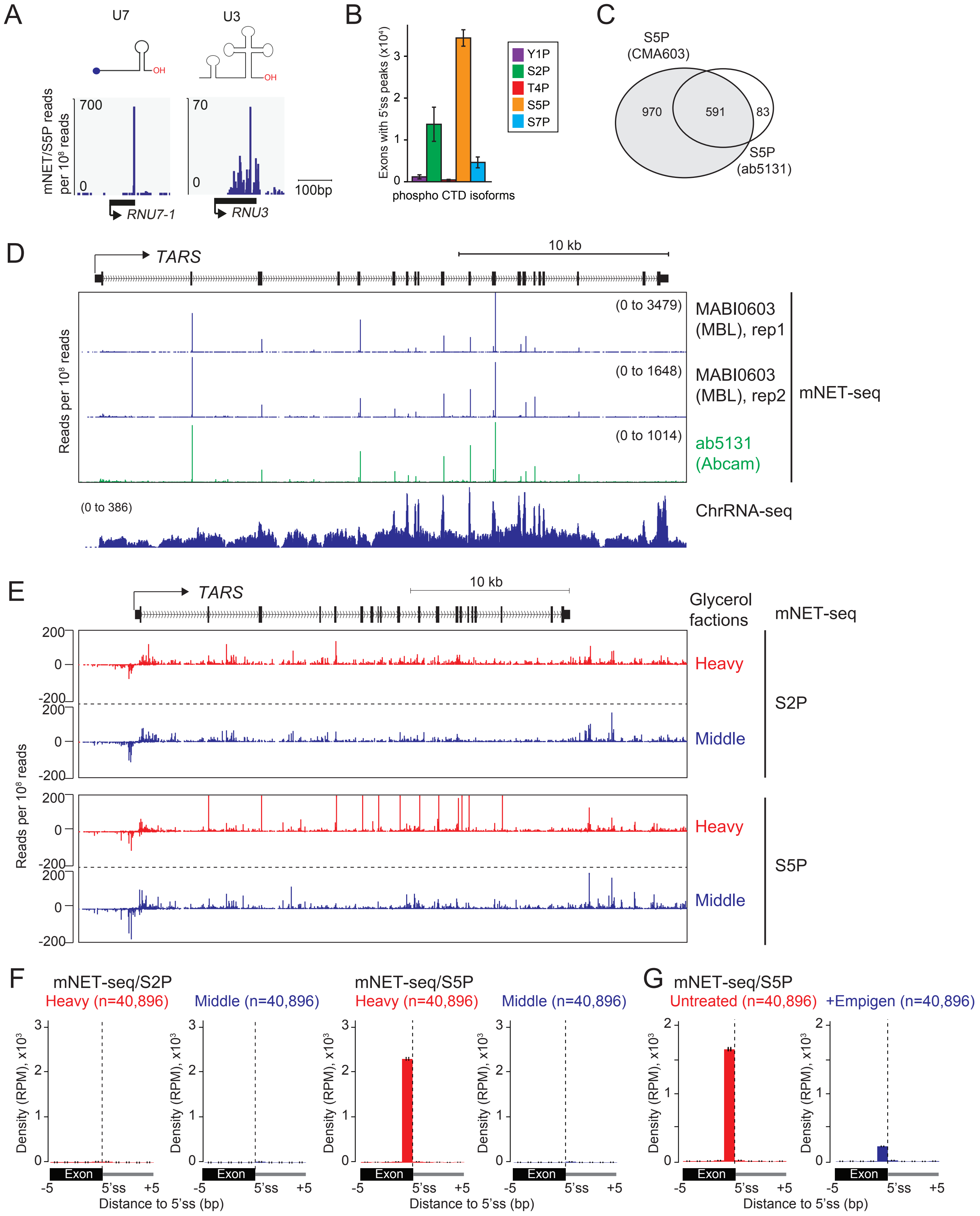


**Figure S2. The catalytically active spliceosome associates with S5P CTD Pol II (related to Figures 1 and 2).**
A) Enlarged mNET-seq profiles over the U7 and U3 snRNA genes using S5P CTDantibody.
B) Number of exons with a 5'ss peak detected by antibodies specific for the indicated CTD modifications (for each antibody, data was obtained from three independent libraries prepared from short RNA fragments).
C) Comparison (Venn diagram) of exons with a 5'ss peak detected using two different S5P CTD specific antibodies; MABI0603 (MBL international) and ab5131 (Abcam)
D) Comparison of mNET-seq/S5P profile over TARS using either MABI0603 (MBL international) or ab5131 (Abcam) antibodies.
E) mNET-seq/S5P and S2P profiles over TARS from heavy and middle glycerol gradient fractions.
F) Metagene analyses of mNET-seq/S5P and S2P profiles at the 5'ss of spliced events (identified in nucleoplasmic RNA-seq datasets) from heavy and middle glycerol gradient fractions.
G) Metagene analyses of mNET-seq/S5P profiles at the 5'ss of spliced events (identified innucleoplasmic RNA-seq datasets) from untreated and empigen-treated samples.

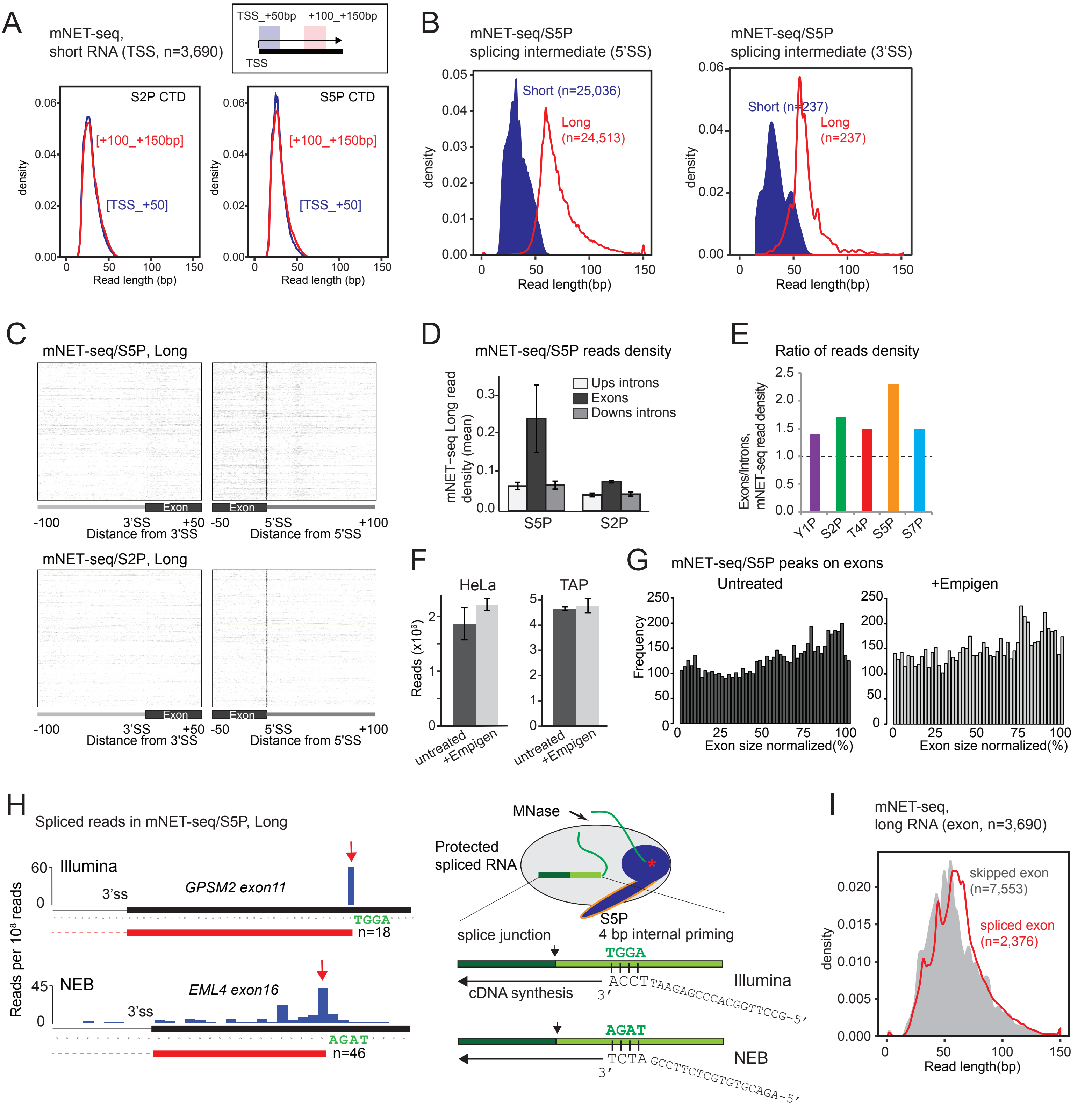


**Figure S3. Elongating RNA Pol II footprints (related to Figure 3).**

A) Length of mNET-seq/S5P and S2P reads mapping within either the first 50 nts or between nts 100 and 150 for 3,690 exons with length >200 nts (data from three independent libraries prepared from short RNA fragments).

B) Length of reads corresponding to 5'ss and 3'ss splicing intermediates (data from three independent libraries prepared from short and long RNA fragments).

C) Heatmaps of mNET-seq/S5P and S2P long RNA reads around 3'ss and 5'ss

D) Quantification of density of mNET-seq/S5P and S2P Long RNA reads on upstream (Ups) intron, exon and downstream (Downs) intron.

E) Ratio of mNET-seq density on exons and introns on all CTD phosphorylation isoforms

F) Quantification of mNET-seq/S5P reads of HeLa and TAP cells on exons with or without empigen treatment.

G) Frequency of mNET-seq/S5P peaks on normalized HeLa exons with or without empigen treatment.

H) Representative examples of 3' ends of spliced reads from libraries prepared with Illumina or NEB cDNA primers. Red arrow denotes position where most 3' ends map. Specific sequence present downstream of this position (as indicated) corresponds to the 3' ends of either Illumina or NEB cDNA primers. Diagram (on the right) illustrating internal priming of spliced transcripts associated with S5P CTD Pol II.

I) Length of reads mapping within included and skipped exons (data from three independent mNET-seq/S5P libraries prepared from long RNA fragments). Reads mapping to the last 3 nts of each exon were excluded to avoid contamination with 5'ss splicing intermediates.

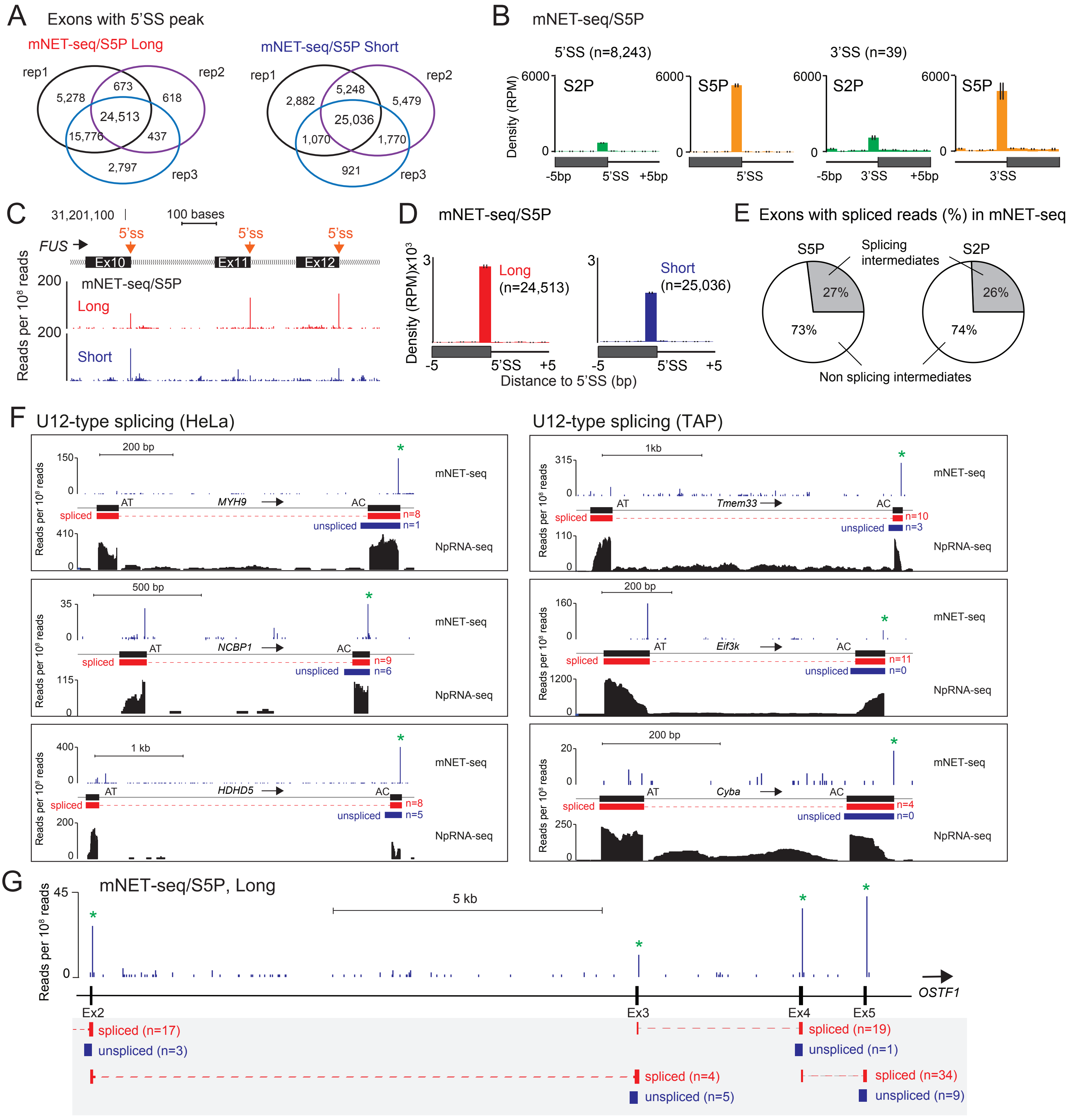


**Figure S4. Sequential co-transcriptional splicing (related to Figure 4).**

A) Venn diagrams showing exons with 5'ss peaks in three independent mNET-seq/S5P libraries prepared from long and short RNA fragments.

B) Metagene analysis of 5'ss and 3'ss peaks detected simultaneously in mNET-seq/S5P and mNETseq/S2P libraries.

C) mNET-seq/S5P profiles over the exon 10-12 region of FUS. Comparison of profiles obtained in libraries prepared with long and short RNA fragments.

D) Metagene analysis of 5'ss peaks detected in mNET-seq/S5P libraries prepared from long and short RNA fragments.

E) Pies depict the proportion of spliced exons associated with 5'ss splicing intermediates (data from three independent mNET-seq/S5P and S2P libraries prepared from long RNA fragments). Only events covered by a minimum of 3 spliced reads were considered.

F) Examples of 5'ss splicing intermediates with U12-type splicing in HeLa (left) and TAP cells (right). Data from three mNET-seq/S5P libraries prepared from long RNA fragments.

G) OSTF1 as an example of sequential splicing. Number of spliced and unspliced reads mapping to the 5'ss downstream of the indicated exons is shown.
