# Supplementary material for: RNA Polymerase II Phosphorylated on CTD Serine 5 Interacts with the Spliceosome during Co-transcriptional Splicing
Source: Mol Cell. 2018 Oct 18;72(2):369–379.e4. doi: 10.1016/j.molcel.2018.09.004 (PMC6201815; doi:10.1016/j.molcel.2018.09.004)
Supplement: Document S2. Article plus Supplemental Information [file mmc3.pdf]

Short Article

Molecular Cell

RNA Polymerase II Phosphorylated on CTD Serine 5 Interacts with the Spliceosome during Co-transcriptional Splicing

Graphical Abstract

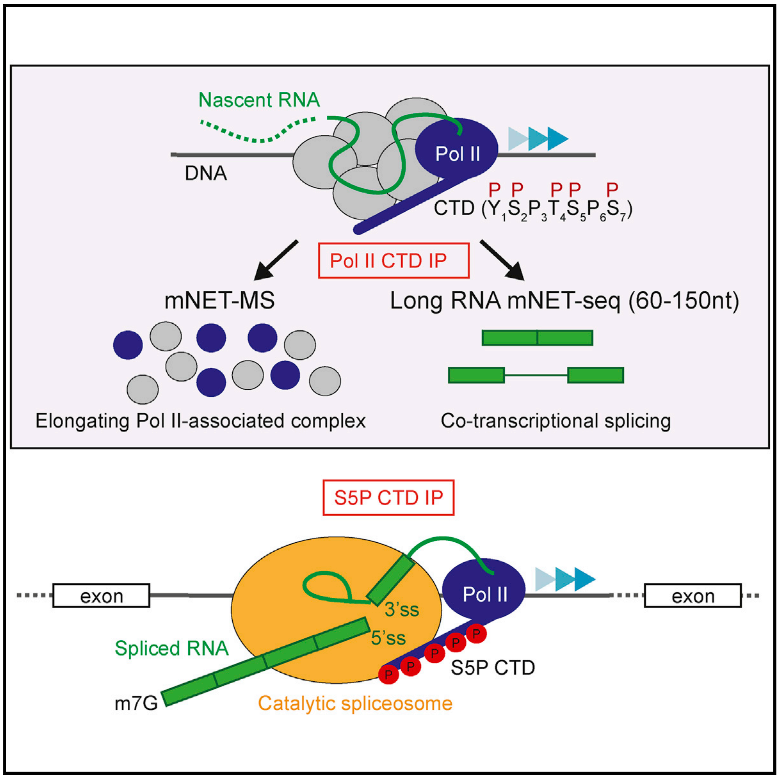

Highlights

- S5P CTD Pol II associates with the catalytic spliceosome
- Elongating Pol II complexes protect about 60 newly synthesized nucleotides
- Co-transcriptional splicing associated with dominant 5′ ss intermediate
- U12-dependent introns are sequentially spliced in association with Pol II

Authors

Takayuki Nojima, Kenny Rebelo, Tomás Gomes, Ana Rita Grosso, Nicholas J. Proudfoot, Maria Carmo-Fonseca

Correspondence

nicholas.proudfoot@path.ox.ac.uk (N.J.P.),
carmo.fonseca@medicina.ulisboa.pt (M.C.-F.)

In Brief

Nojima and colleagues demonstrate widespread co-transcriptional splicing of mammalian genes by complex formation between the active spliceosome and the RNA polymerase II S5P CTD isoform. RNA processing intermediates within this complex are invariably detected with the transcript cleaved at 5′ ss but already spliced to upstream exons, implying a sequential splicing process.

Nojima et al., 2018, Molecular Cell *72*, 369–379
October 18, 2018 © 2018 The Authors. Published by Elsevier Inc.
https://doi.org/10.1016/j.molcel.2018.09.004

RNA Polymerase II Phosphorylated on CTD Serine 5 Interacts with the Spliceosome during Co-transcriptional Splicing

Takayuki Nojima,[1,4] Kenny Rebelo,[2,4] Tomás Gomes,[2,3] Ana Rita Grosso,[2] Nicholas J. Proudfoot,[1,5,*] and Maria Carmo-Fonseca[2,*]
[1]Sir William Dunn School of Pathology, University of Oxford, South Parks Road, Oxford OX1 3RE, United Kingdom
[2]Instituto de Medicina Molecular, Faculdade de Medicina, Universidade de Lisboa, 1649-028 Lisboa, Portugal
[3]Wellcome trust Sanger Institute, Wellcome Genome Campus, Hinxton, Cambridge, CB10 1SA, United Kingdom
[4]These authors contributed equally
[5]Lead Contact
*Correspondence: nicholas.proudfoot@path.ox.ac.uk (N.J.P.), carmo.fonseca@medicina.ulisboa.pt (M.C.-F.)
https://doi.org/10.1016/j.molcel.2018.09.004

SUMMARY

The highly intronic nature of protein coding genes in mammals necessitates a co-transcriptional splicing mechanism as revealed by mNET-seq analysis. Immunoprecipitation of MNase-digested chromatin with antibodies against RNA polymerase II (Pol II) shows that active spliceosomes (both snRNA and proteins) are complexed to Pol II S5P CTD during elongation and co-transcriptional splicing. Notably, elongating Pol II-spliceosome complexes form strong interactions with nascent transcripts, resulting in footprints of approximately 60 nucleotides. Also, splicing intermediates formed by cleavage at the 5′ splice site are associated with nearly all spliced exons. These spliceosome-bound intermediates are frequently ligated to upstream exons, implying a sequential, constitutive, and U12-dependent splicing process. Finally, lack of detectable spliced products connected to the Pol II active site in human HeLa or murine lymphoid cells suggests that splicing does not occur immediately following 3′ splice site synthesis. Our results imply that most mammalian splicing requires exon definition for completion.

INTRODUCTION

Eukaryotic protein coding genes often contain multiple introns that are removed from primary transcripts of RNA polymerase II (Pol II) by splicing. This is catalyzed by the spliceosome, comprising five snRNAs and more than 200 proteins, a subset of which form functional ribonucleoprotein particles. Exon-intron boundaries are defined by short consensus sequences at the 5′ and 3′ splice sites (ss) that mediate recognition by the spliceosome. Spliceosome assembly also requires recognition of an intronic catalytic adenosine (branch point) and, for mammalian introns, a polypyrimidine tract located between the branch point adenosine and 3′ ss. Within the 3D structure of the spliceosome, these key pre-mRNA sequences are forced into an RNA catalytic center that triggers splicing (Papasaikas and Valcárcel, 2016). This involves two transesterification reactions. First, the 2′ hydroxyl group (OH) of the branch point adenosine carries out a nucleophilic attack on the phosphate group at the 5′ ss; then the exposed 3′ OH of the upstream exon cleaves the lariat structured intron away from the 3′ ss, resulting in covalent joining of the two exons (Patel and Steitz, 2003).

Exactly how two splice sites separated by extensive intronic sequence (in mammals, 1 to 100 kb) are brought together during early stages of spliceosome assembly remains a puzzle. Possibly, the 5′ exon is tethered to elongating Pol II until complete synthesis of the downstream intron has occurred. This would maintain the two splice sites in close proximity, irrespective of intron size (Hollander et al., 2016). Co-transcriptional tethering of the 5′ exon may involve the flexible C-terminal domain (CTD) of Pol II's largest subunit, Rbp1, which acts to coordinate transcription with pre-mRNA processing (Heidemann et al., 2013; Zaborowska et al., 2016). Mammalian CTD comprises 52 repeats of a heptad with the consensus amino acid sequence YSPTSPS. This is extensively modified post-translationally, principally by phosphorylation to S2P, S5P, S7P, T4P, and Y1P (Hintermair et al., 2012; Hsin and Manley, 2012; Mayer et al., 2012). At the promoter, Pol II CTD is largely unphosphorylated, but then, following initial elongation, it is converted to S5P and Y1P. This may facilitate recruitment of the capping enzyme complex. Further elongation correlates with a reduction in S5P but an increase in S2P. Transcription past the polyadenylation site then triggers recruitment of the 3′ end cleavage and polyadenylation machinery to S2P. It is possible that T4P also plays a role here, as it has recently been correlated with Pol II termination regions (Hintermair et al., 2012; Schlackow et al., 2017).

The kinetics of co-transcriptional splicing in budding yeast has been extensively characterized (Wallace and Beggs, 2017). The short size of yeast (*S. cerevisiae*) introns implies an intron definition model for the splicing mechanism (Ast, 2004; Barrass and Beggs, 2003; Chen and Manley, 2009). In particular, Pol II pausing has been observed over the 3′ ss of downstream exons in the few genes that harbor introns. This may facilitate rapid

© 2018 The Authors. Published by Elsevier Inc.
This is an open access article under the CC BY license (http://creativecommons.org/licenses/by/4.0/).

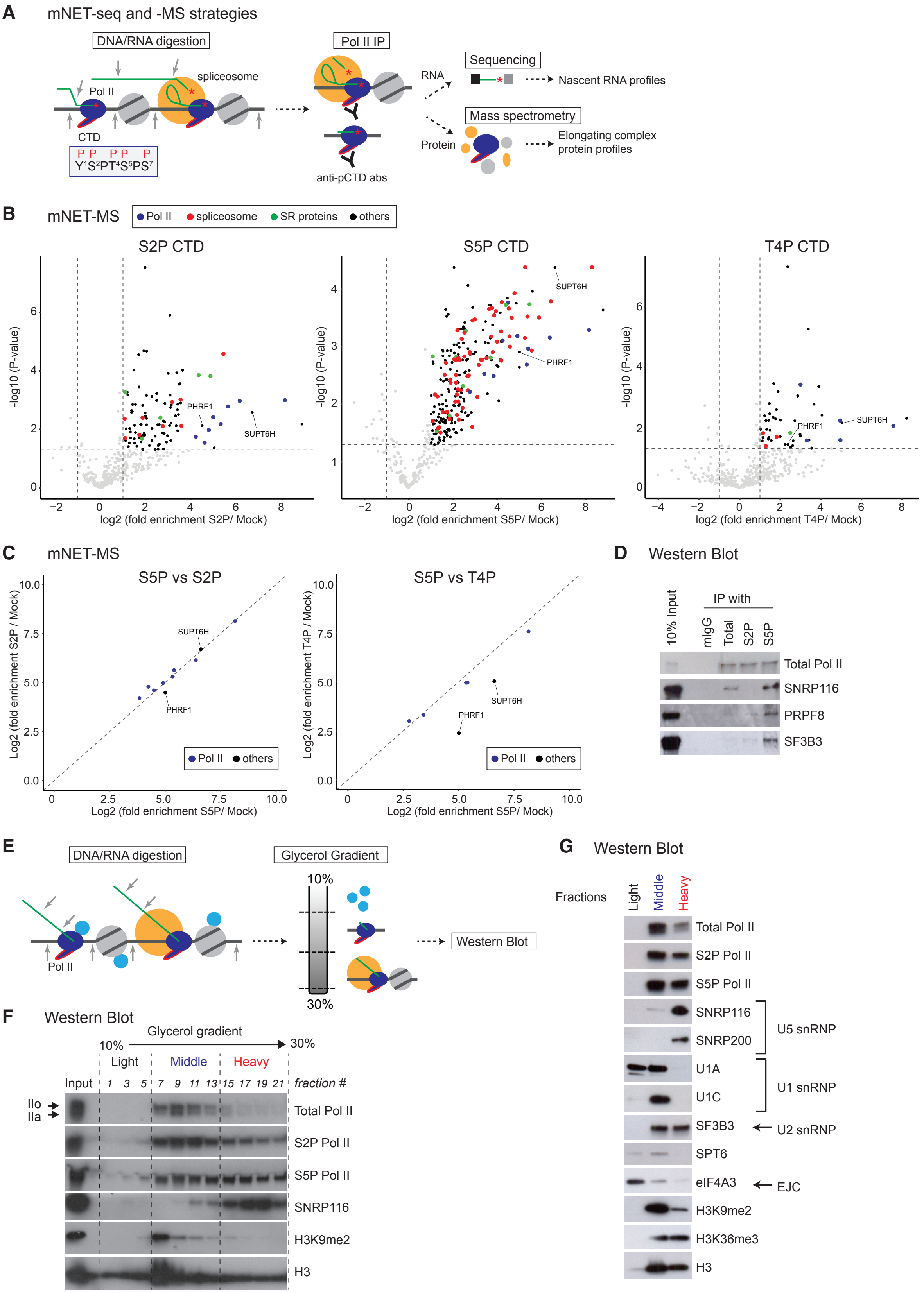

(legend on next page)

spliceosome recruitment and intron splicing (Alexander et al., 2010). Furthermore, splicing in *S. cerevisiae* has been shown to occur within a short kinetic window following Pol II elongation through an intron and into the adjoining exon (Oesterreich et al., 2016). In mammals, the prevalence and larger size of introns instead predicts an exon definition mechanism. Binding of regulatory proteins to specific RNA sequences (splicing enhancers and inhibitors) act to select exons for splicing by promoting communication between 3′ ss and 5′ ss flanking the exon (Ast, 2004; Chen and Manley, 2009). The interplay between these regulatory factors dictates both constitutive and alternative splicing. Notably, transcription kinetics strongly impact splicing decisions. Slow Pol II elongation rates favor splicing by allowing more time for spliceosome assembly (Dujardin et al., 2014; Muñoz et al., 2010). Exons in general display a higher nucleosome density, suggesting that chromatin structure acts as a kinetic barrier to Pol II elongation (Nieto Moreno et al., 2015; Schwartz and Ast, 2010).

We have developed a strategy for native elongation transcript sequencing using mammalian cells (mNET-seq). We found an accumulation of transcripts mapping precisely to the 3′ end of exons, as expected for intermediates formed after the first transesterification splicing reaction (Nojima et al., 2015). This indicates that splicing must occur within a stable complex formed between the spliceosome and Pol II. Unexpectedly, splicing intermediates were preferentially detected by antibodies specific for S5P CTD, suggesting that splicing occurs in association with this Pol II phospho-isoform. We now show biochemically and by mass spectroscopy that both protein and snRNA components of the spliceosome associate with Pol II complexes containing S5P CTD. Notably, initial cleavage of the intron creates a dominant 5′ ss intermediate that remains embedded in the Pol II-associated spliceosome. In contrast, 3′ ss intermediates corresponding to released intron lariats were detected at very low levels, suggesting fast dissociation of the spliceosome from Pol II upon completion of splicing.

RESULTS

Catalytically Active Spliceosome Associates with S5P CTD Pol II

mNET-seq involves immunoprecipitation (IP) of human Pol II elongation complexes from chromatin solubilized by micrococcal nuclease (MNase) digestion with Pol II antibodies specific for different CTD isoforms (Nojima et al., 2015; Schlackow et al., 2017). RNA is isolated from these complexes and sequenced by linker ligation onto the RNA 3′ ends derived either from nascent RNA in the Pol II active site or co-transcriptional RNA processing intermediates. Notably, we showed that co-transcriptional splicing is associated with Pol II phosphorylated on the CTD serine 5 position (S5P) (Nojima et al., 2015; Schlackow et al., 2017). We reasoned that, as well as sequencing IPed RNA, we should also be able to establish the protein composition of these specifically IPed Pol II complexes by mass spectroscopy (MS), termed the mNET-MS method (Figure 1A). A label-free quantitative proteomics approach (Hubner et al., 2010; Hubner and Mann, 2011) was used to determine the abundance of proteins enriched in Pol II complexes IPed with antibodies specific for S5P, S2P, and T4P CTD relative to a mock (nonspecific IgG) IP control. The fold increase of spectral intensities was compared to p values determined by t tests, as previously described (Harlen et al., 2016), generating volcano plots (Figure 1B). Significant interactors were taken to be proteins identified as enriched using a false discovery rate of 0.05. We show that complexes precipitated by all three antibodies contained Pol II subunits, transcription factors, histones, chromatin-associated proteins, RNA-binding proteins, and RNA-processing factors (Table S1). Scatterplots with fold-enrichment reveal that Pol II subunits were precipitated with similar efficiency to SUPT6H and PHRF1 (Figure 1C). Remarkably, complexes precipitated by the S5P CTD antibody contained multiple SR and SR-related proteins (SRSF7, 9,10; SCAF1, 11), as well as spliceosome components. These include Sm proteins, U1 and U2 snRNP-specific proteins, and components of spliceosomal A, B, Bact, and C complexes (Figures 1B and S1; Table S1). This was confirmed by western blot analysis of fractions IPed with S5P compared to S2P antibodies; S5P-specific enrichment was observed for the U5 snRNP protein, SNRP116, the splicing scaffold protein, PRPF8, and the U2 snRNP protein SF3B3 (Figure 1D). There are several possible reasons for this selectivity. Either spliceosomal components interact specifically with S5P CTD or, possibly, S5P and S2P epitopes display differential antibody accessibility. However, we consider this later possibility unlikely, as a recent proteomic analysis in yeast also revealed an enrichment of spliceosomal components in S5P Pol II complexes (Harlen et al., 2016).

As an alternative approach to studying the interaction between Pol II and the spliceosome, chromatin was solubilized by MNase treatment and then fractionated on a 10%–30% glycerol gradient followed by protein extraction from 22 separated fractions (Figure 1E). These were subjected to western blotting with antibodies against total, S2P, and S5P Pol II as well as U5

Figure 1. Spliceosome Proteins Associate with S5P CTD Pol II

(A) Diagram outlining mNET-seq and associated mass spectroscopy strategies. Pol II denoted in dark blue with CTD tail and heptad repeat sequence modified by phosphorylation (P) indicated in red edging. Nucleosomes shown in gray and spliceosome in orange. Grey arrows denote micrococcal nuclease cleavage and red asterisks indicate free 3′ OH at the end of the nascent transcript and cleaved 5′ ss splicing intermediate.

(B) Volcano plots comparing S2P, S5P, and T4P CTD Pol II IPs to mock IPs. Specifically, enriched proteins are highlighted in black and color coded (see Table S1 for identification of all enriched proteins).

(C) Scatterplots of fold enrichment for Pol II subunits (highlighted in blue), SUPT6H, and PHRF1 in S5P versus S2P IPs and S5P versus T4P IPs.

(D) Western blots with indicated antibodies of micrococcal nuclease-solubilized HeLa cell chromatin before (input) and after immunoprecipitation (IP) with either no specific antibody (mIgG) or antibodies to total S2P and S5P CTD Pol II.

(E) Diagram outlining the glycerol gradient fractionation of micrococcal nuclease-solubilized chromatin.

(F) Western blots across glycerol gradient fractions.

(G) Western blots on pooled light, middle, and heavy fractions from glycerol gradient.

snRNP protein SNRP116 and histone H3 (Figure 1F). Significant amounts of S2P, S5P Pol II, and histone H3 were detected in the middle and heavy fractions. However, the heavy fraction contained most of the snRNP116 (Figure 1F). Further analysis with a wider range of antibodies against pooled light, middle, and heavy fractions confirms that U5 snRNP proteins were predominantly associated with the heavy fraction (Figure 1G). In contrast, U2 snRNP proteins appeared similarly distributed in the middle and heavy fractions, and U1 snRNP proteins were predominantly associated with the middle fraction (Figure 1G). The histone mark H3K9me2, which is typically associated with transcriptional repression, was predominantly in the middle fraction, whereas the histone elongation mark H3K36me3 was in both the middle and heavy fractions. These results indicate that the heavy fraction contains spliceosome-associated S5P Pol II-elongating polymerases on a chromatin template.

Since S5P CTD modified Pol II interacts with protein components of the spliceosome, we also analyzed spliceosomal snRNA. We reasoned that mNET-seq should detect the free 3′ OH ends of mature spliceosomal snRNA associated with Pol II. Indeed, an accumulation of mNET-seq signal was found mapping to the 3′ ends of U1, U2, U4, and U5 snRNAs (Figure 2A). As expected, no 3′ end peak was detected in U6 snRNA, which contains a 2′,3′ cyclic phosphate terminal group at the 3′ end (Lund and Dahlberg, 1992). Lower mNET-seq peaks were also found at the 3′ ends of the minor spliceosome U11, U12, and U4atac snRNAs, but not in U6atac snRNA (Figure 2B). A weak 3′ end signal was further detected for U7 snRNA, which is associated with histone pre-mRNA 3′ end processing. As expected, no 3′ end peak was detected for U3 snRNA, which is involved in the processing of Pol I-synthesized pre-rRNA (Figures 2C and S2A). To confirm the specificity of these results, we tested whether the spliceosome snRNAs' 3′ end signal was sensitive to treatment with the strong detergent empigen. We have previously employed empigen to separate RNA processing complexes from Pol II (Schlackow et al., 2017). Notably mNET-seq signals of both major and minor spliceosome snRNAs 3′ ends were substantially diminished (Figure 2D). A more detailed comparison of the association of mature spliceosomal snRNA (based on mNET-seq 3′ end reads) for all phospho CTD isoforms of Pol II revealed higher signal with S5P CTD antibody for U2, U5, and the minor spliceosome snRNA. In contrast, U1 snRNA showed similar read levels for all five phospho CTD isoforms (Figure 2E).

To complete this mNET-seq analysis of spliceosome-associated RNA, we also tested the distribution of the 5′ ss splicing intermediate. We first confirmed that RNA fragments mapping to the 5′ ss are predominantly detected by S5P CTD antibody as opposed to S2P, Y1P, T4P and S7P antibodies (Figure S2B). We also compared mNET-seq profiles with different CTD S5P-specific antibodies; MABI0603 (MBL international), routinely used in our mNET-seq/S5P analysis, and ab5131 (Abcam), often used in Pol II ChIP analysis. Similar profiles of 5′ ss peaks were obtained (Figures S2C and S2D), consistent with our previous ChIP analysis that showed their same specificity for S5P CTD (Nojima et al., 2015). We next generated mNET-seq/S5P and S2P libraries from the heavy and middle fractions of the glycerol gradient of MNase-digested chromatin. Notably, the 5′ ss splicing intermediate was only detected from the heavy S5P library as shown for *TARS* (Figure S2E) and in a metagene profile of 40,896 aligned exon intron junctions corresponding to spliced events (Figure S2F). These results confirm the existence of a complex between Pol II and the spliceosome in heavy fractions. Finally, since the 5′ ss splicing intermediate detected by mNETseq/S5P is empigen sensitive (Figure S2G), we predict that the 5′ ss RNA is associated with the spliceosome rather than with the Pol II active site.

In summary, these data strongly argue that phosphorylation of CTD serine 5 residues is not restricted to transcription initiation but is also present during elongation and co-transcriptional splicing. It is clear from the range of data presented (Figures 1, 2, S1, and S2) that catalytically active spliceosome stably associates with S5P CTD Pol II.

mNET-Seq Reveals Elongating RNA Pol II Footprints

We next employed mNET-seq to investigate the extent of nascent transcript interaction with both Pol II and the spliceosome. Since this procedure involves MNase digestion before RNA purification and library preparation, it can be used for Pol II and spliceosome footprinting analysis akin to ribosome footprinting. Previously, we size selected RNA of 30–100 nt for library preparation (Nojima et al., 2015; Schlackow et al., 2017). To explore a larger RNA size range, we prepared new mNETseq/S5P libraries using long-sized (60–160 nt) or short-sized (20–60 nt) RNA fractions with a read length of 150 bp and 75 bp, respectively (Figure 3A). To analyze Pol II footprints independently of spliceosome assembly, we examined the length of reads mapping to a region from the TSS to +150 nucleotides in first exons that were at least 200 nt long. Most reads (> 85%) obtained from the short-sized RNA fractions were 18–40 nt long with a peak at 27 nt, whereas those for the long-sized RNA fractions were 40–100 nt long with a peak at 60 nt (Figure 3B). We infer that Pol II complexes protect a total of either 27 or 60 nascent transcript nucleotides. Previous structural data indicate that nascent RNA must be at least 15 nt in length to reach the Pol II surface (Andrecka et al., 2009; Kettenberger et al., 2004) and 17–23 nt to reach the active site of the capping enzyme, which binds Pol II at the end of the RNA exit tunnel (Martinez-Rucobo et al., 2015). To determine whether binding of the capping enzyme to Pol II explains the 20–30 nt footprint, we compared the length of reads mapping to the same exons on the region from the TSS to +50 nts and from +100 to +150 nts (Figure S3A). A similar footprint was obtained irrespective of read position with mNET-seq libraries prepared after IP with either S5P and S2P antibodies (Figure S3A). This suggests that once nascent transcripts emerge from the polymerase exit channel, they are protected from MNase by RNA-binding proteins such as the capping enzyme. The larger 60-nt footprint obtained from long-sized RNA fractions (Figure 3B) was further characterized. Analysis of mNET-seq profiles obtained from the long-sized RNA fractions no longer shows the characteristic peaks corresponding to promoter proximal transcriptional pausing that typically occurs within the first 50 bp of the transcription start site (TSS). This is shown both for the TSS region of *DAP3* (Figure 3C) and a metagene profile of long and short mNET-seq/S5P TSS reads (Figure 3D). We conclude that the mNET-seq protocol distinguishes two types of elongating Pol II complexes, a smaller one corresponding to a nascent RNA footprint of 27 nt and a larger one protecting approximately 60 nt of nascent transcripts (Figure 3E).

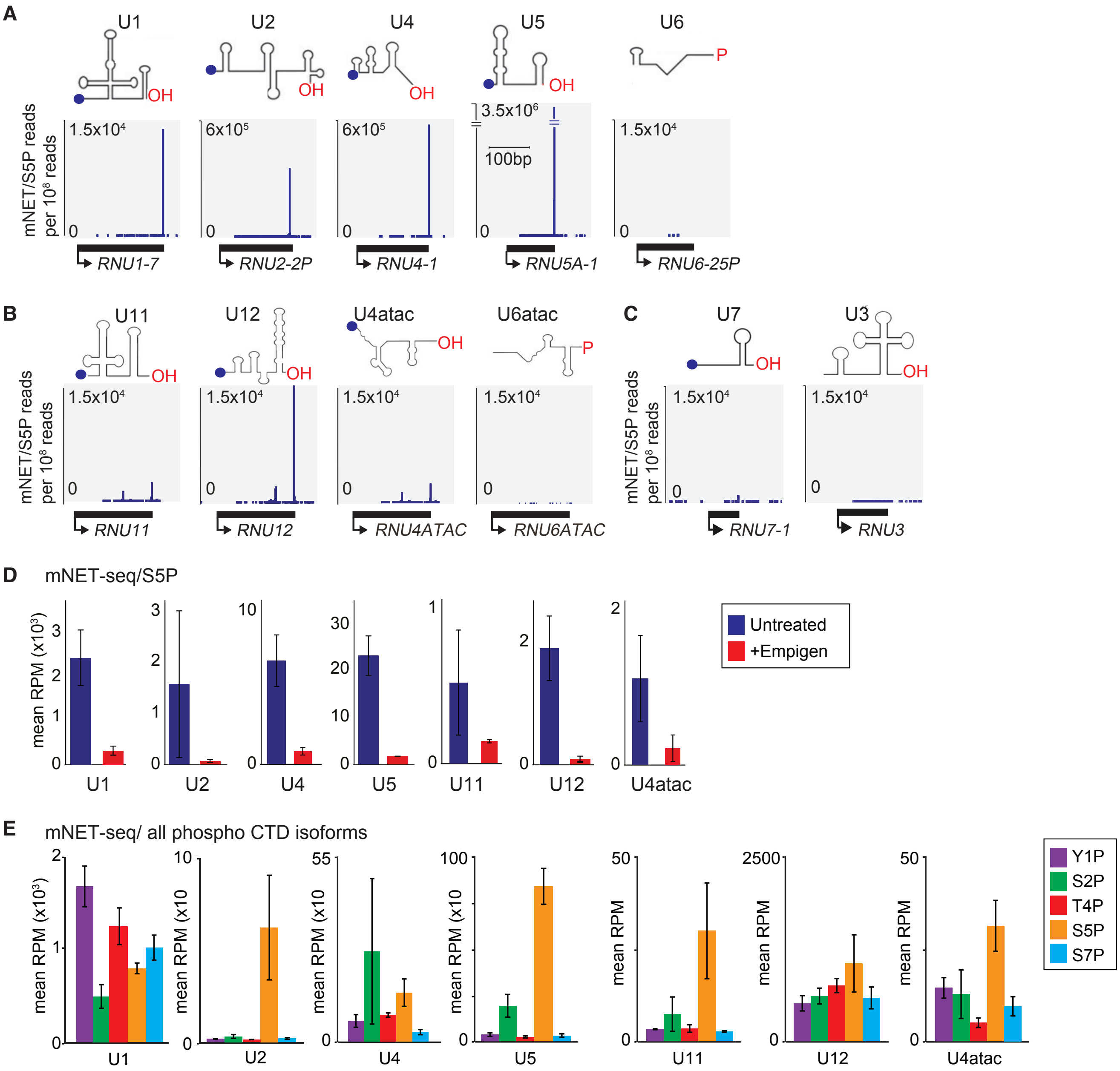

Figure 2. Spliceosome snRNAs Associate with S5P CTD Pol II

(A–C) mNET-seq/S5P profiles over indicated snRNA genes: major spliceosome snRNAs (A); minor spliceosome snRNAs (B); non-spliceosome snRNAs (C).

(D) Aggregated read counts (mean RPM) mapping to indicated snRNA genes in untreated and empigen-treated samples (data from three independent mNET-seq/S5P libraries). Data are represented as mean ± SEM.

(E) Aggregated read counts (mean RPM) obtained with antibodies to color-coded CTD modifications and mapping to indicated snRNA genes (data from three independent mNET-seq libraries with each CTD antibody). Data are represented as mean ± SEM.

Next, we examined the read length of splicing intermediates in mNETseq/S5P and S2P libraries prepared from the short and long-sized RNA fractions (Figure S3B). The results show that the most frequent length of both 5′ ss intermediates (i.e., exons that have been cleaved at the 5′ ss but not yet ligated) and 3′ ss intermediates (i.e., released intron lariats) is 30–32 nt in the short libraries and 57–60 nt in the long libraries. As splicing intermediates are spliceosome-bound, we conclude that these fragments result from the footprinting of splicing complexes associated with S5P and S2P CTD Pol II. Notably, a very recent analysis of RNA co-purifying with late-stage spliceosomes similarly revealed that splicing complexes protect a total of approximately 56 nucleotides (Chen et al., 2018).

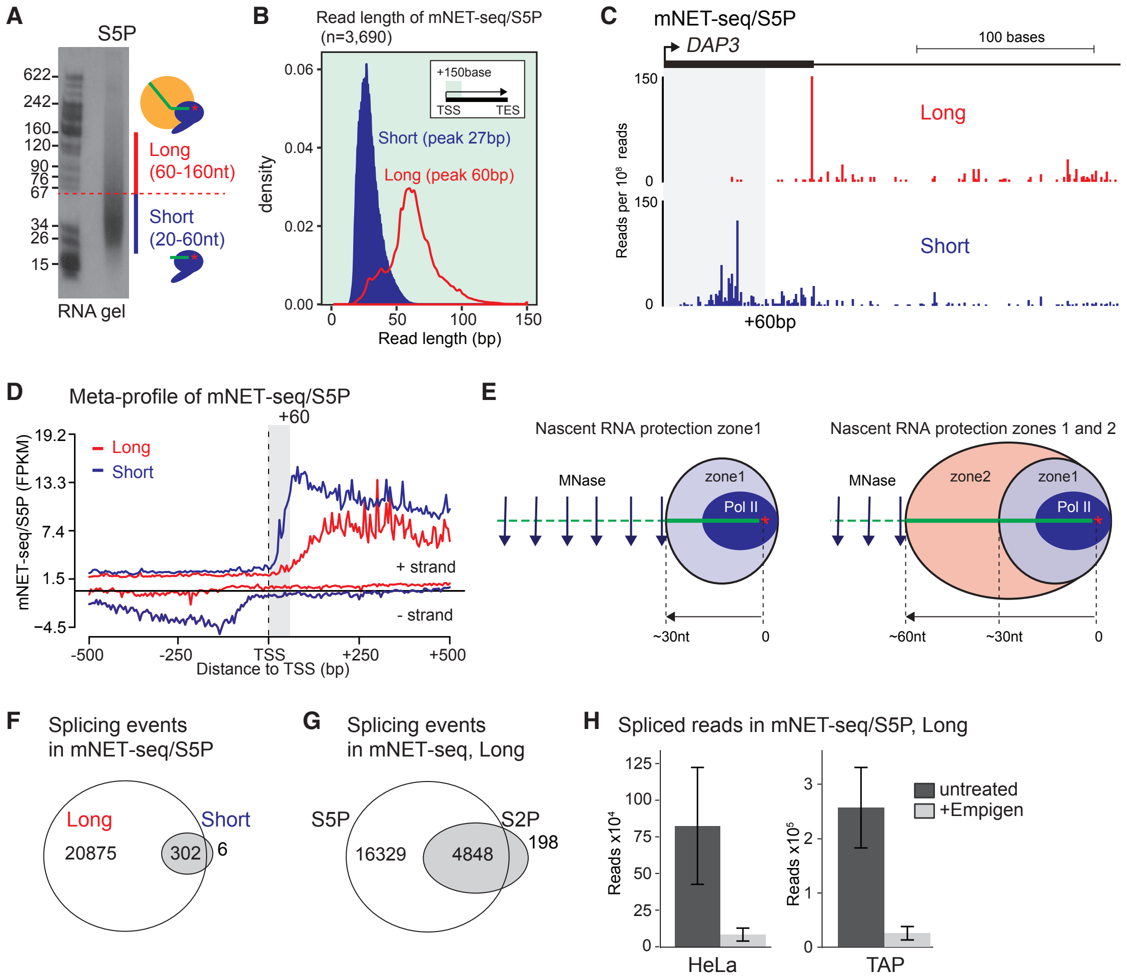

Figure 3. mNET-seq reveals elongating RNA Pol II footprints

(A) Size selection of RNA for different mNET-seq/S5P libraries.

(B) Length of reads mapping within the first 150 nts for 3,690 exons with length > 200 nts.

(C) mNET-seq/S5P profiles over the promoter-proximal region of *DAP3*. Comparison of profiles from libraries prepared with long and short RNA fragments.

(D) Metagene analysis of mNET-seq/S5P profiles over the promoter-proximal region from libraries prepared with long and short RNA fragments.

(E) Diagram depicting footprints of Pol II complexes on short and long nascent RNA fragments in micrococcal nuclease digested chromatin.

(F) Venn diagram comparing splicing events covered by at least three spliced reads in three independent mNET-seq/S5P libraries prepared from long and short RNA fragments.

(G) Venn diagram comparing splicing events covered by three or more spliced reads in three independent mNET-seq/S5P and S2P libraries prepared from long RNA fragments.

(H) Aggregated spliced read counts obtained in HeLa and TAP cells with or without Empigen treatment (data from three independent mNET-seq/S5P libraries prepared from long RNA fragments). Data are represented as mean ± SEM.

mNET-Seq Does Not Detect Spliced Nascent Transcripts

We show above (Figures 1 and 2) that the spliceosome forms a complex with Pol II. We therefore tested if splicing can occur immediately after the 3′ ss in a nascent transcript emerges at the Pol II surface. We reasoned that, if this was the case, then mNET-seq should detect spliced nascent transcripts. Analysis of mNET-seq libraries prepared from the short RNA fraction revealed spliced exon reads corresponding to approximately 300 splicing events, while over 20,000 splicing events were detected in libraries prepared from the long RNA fraction (Figure 3F). Most spliced events detected by spliced exon reads were predominantly observed in mNET-seq/S5P libraries (Figure 3G), consistent with our data indicating that complexes of Pol II and associated catalytically active spliceosomes are preferentially IPed by S5P CTD antibodies (Figures 1, 2, S1, and S2). At this stage in our analysis, we added additional mNET-seq/S5P libraries for the mouse lymphoid cell line TAP to generalize our results on co-transcriptional splicing to a different mammal and cell type. We also performed heat-map analysis of mNET-seq signals and detected more S5P signals on spliced exons than other phospho CTD isoforms (Figures

S3C–S3E). Notably these mNET-seq/S5P signals are resistant to empigen, suggesting that they are derived from nascent transcripts connected to the Pol II active site (Figure S3F). These empigen-resistant mNET-seq/S5P signals do not show any bias across exons (Figure S3G). This indicates that S5P CTD Pol II pausing is exon position independent. In contrast, the vast majority of the spliced reads in human and murine libraries display empigen sensitivity (Figure 3H), showing that they are nascent RNAs associated with Pol II (presumably within the spliceosome) but not from the Pol II active site. The combined analysis of human and murine libraries led us to appreciate that cDNA primers used in next-generation sequencing can internally prime on spliceosome-derived spliced transcripts as well as priming on the linker ligated onto RNA 3′ ends (either 5′ ss intermediates or Pol II active site RNA). This was evident from a conserved 4-nt sequence (TGGA from Illumina TruSeq RT primer) present at the 3′ end of the spliced RNA reads that corresponds to the 3′ end of the cDNA primer (Figure S3H). Even when the RT primer was changed, we still detected the same internal priming issue (AGAT from NEB smRNA RT primer). Overall, these results demonstrate that even though numerous spliced read transcripts are specifically detectable in our mNET-seq/S5P libraries, they do not reflect immediate splicing of transcripts synthesized by a polymerase that has elongated just a few nts past the 3′ ss as seen in *S. cerevisiae* (Oesterreich et al., 2016). Although spliced reads may correspond to contaminating fully spliced mRNA that is detected due to mis-priming, it is unlikely that contaminants would be specifically enriched by S5P antibody. Rather, these spliced reads likely derive from fragments of spliced products connected to Pol-II-associated spliceosomes.

We further reasoned that if a spliceosome assembles onto nascent pre-mRNA as soon as the 3′ ss is exposed at the Pol II surface, akin to the binding of the capping machinery, this should result in extended nascent transcripts fragments protected from MNase digestion. We therefore analyzed the length distribution of reads mapping to included or skipped exons, but we obtained similar footprint sizes (Figure S3I). Taken together, the lack of detectable spliced read transcripts connected to the Pol II active site and the absence of combined Pol II-spliceosome footprints argue that most splicing occurs when Pol II has elongated for a significant distance beyond the 3′ ss. These observations are consistent with the view that most mammalian splicing requires exon definition for successful completion and therefore, unlike yeast, mammalian splicing only occurs following full exon synthesis.

Co-Transcriptional Splicing Is Globally Associated with 5′ ss Intermediates

The ability of mNET-seq to map splicing intermediates enables in-depth investigation of the extent of co-transcriptional splicing *in vivo*. Because the first step of the splicing reaction (i.e., cleavage at the 5′ ss) results in multiple mNET-seq reads mapping to the very last nucleotide of exons, we sought to identify the proportion of exons with 5′ ss peaks. For this we adapted a previously described algorithm that finds nucleotides where the read density is at least three standard deviations above the mean in a defined window (Churchman and Weissman, 2011). Upon analyzing replicates of mNET-seq/S5P long and short RNA libraries, we found substantial overlap of approximately 25,000 exons showing 5′ ss peaks between three independent datasets (Figure S4A). In contrast, 3′ ss peaks that correspond to released intron lariats were only detected at very low levels (up to 170 cases) (Figure 4A). Like 5′ ss peaks, these few cases of 3′ ss peaks were again predominantly detected by the S5P antibody (Figure S4B). We also investigated the intensity of the signal at the end of exons in the long and short mNET-seq/S5P libraries. As shown for *FUS* (Figure S4C) and in a meta gene analysis (Figure S4D), higher 5′ ss peaks were seen for the long library. We next ascertained the proportion of splicing events in the HeLa cell transcriptome that show a 5′ ss intermediate by performing a global analysis of the percent splicing index (PSI). This measures the inclusion level of internal exons based on nucleoplasm-derived RNA-seq (Nojima et al., 2015) and shows that nearly all exons with 5′ ss peaks are fully included in mature mRNAs (PSI > 0.9). Remarkably, 88% of efficiently spliced exons show a 5′ ss mNET-seq peak, indicating that this splicing intermediate is a dominant splicing feature (Figure 4B).

mNET-Seq Reveals Sequential Co-transcriptional Splicing

We suggest above that the majority of mammalian splicing occurs in association with Pol II but with a lag between synthesis of the 3′ ss and assembly of the spliceosome onto the nascent transcript. We next investigated the prevalence of upstream spliced exons covalently connected to 5′ ss splicing intermediates, as this would be indicative of sequential co-transcriptional splicing. In HeLa long-read mNET-seq/S5P libraries, we detected over 4,000 exons with spliced reads mapping to splicing intermediates (Figure 4C). Although these were more frequently detected in mNET-seq/S5P, both S5P and S2P libraries contained approximately one quarter of all spliced exon reads associated with splicing intermediates (Figure S4E). We also detect 5′ ss intermediates associated with spliced reads in TAP long-read mNET-seq/S5P libraries, indicating that both cell types display a predominance of spliced relative to unspliced reads associated with the 5′ ss intermediate (Figure 4D). Thus, when these exons are cleaved at the 5′ ss, their upstream intron has already been spliced (Figure 4E). However, sequential splicing is not an obligatory rule, as we also observe 5′ ss intermediates associated with unspliced reads, particularly in the case of alternatively spliced exons (Figure 4F). Remarkably, the rarer examples of minor U12-type introns also show a predominance for spliced over unspliced 5′ ss intermediates in both HeLa and TAP cells (Figures 4G and S4F). Although previous studies suggested that U12-associated splicing is less efficient than splicing of U2-type introns (Turunen et al., 2013), our results demonstrate that U12-type introns have been excised by the time a spliceosome initiates splicing of the downstream exons.

DISCUSSION

We have previously described a dominant splicing intermediate generated by the first catalytic step in splicing, which is associated with S5P CTD-modified Pol II (Nojima et al., 2015). This suggested that cleaved upstream exons are retained on the

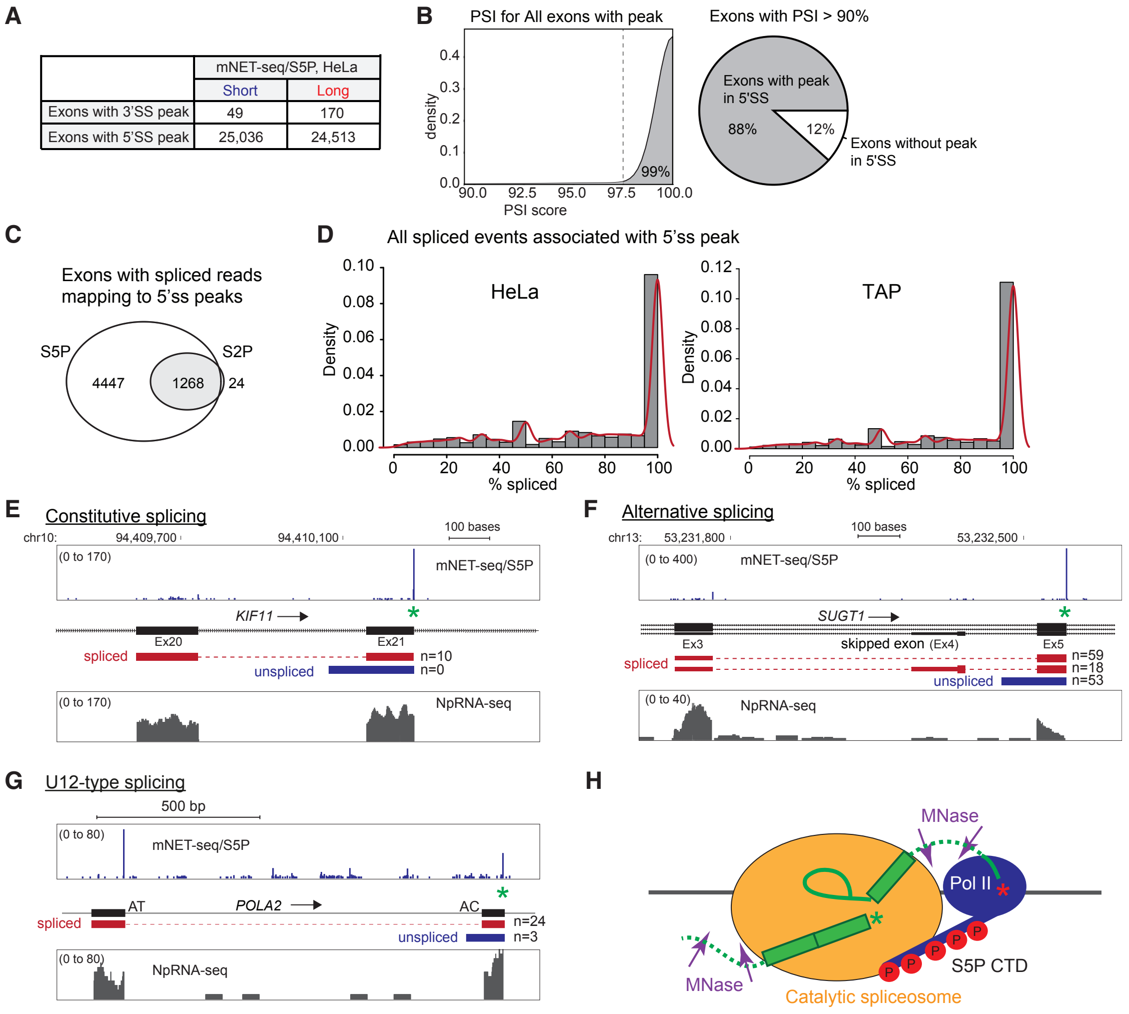

Figure 4. mNET-Seq Reveals Sequential Co-transcriptional Splicing

(A) Number of exons with a peak of mNET-seq/S5P signal detected at either the 5′ ss or the 3′ ss (only peaks detected in all three libraries prepared with long and short RNA fragments were considered).

(B) Percent spliced in (PSI) score for all exons containing a peak of mNET-seq/S5P signal at 5′ ss (aggregated data from three libraries). Pie chart depicts the proportion of spliced exons (exons with PSI > 90% estimated from nucleoplasmic RNA-seq data) that contain a peak of mNET-seq/S5P signal at the 5′ ss (aggregated data from three libraries).

(C) Venn diagram comparing spliced 5′ ss intermediates detected in three independent mNETseq/S5P and mNET-seq/S2P libraries prepared with long RNA fragments.

(D) Ratio between spliced and unspliced reads for all splicing events associated with at least one spliced read mapping to the 5′ ss intermediate. Graphs depict frequency of events with indicated proportions of spliced reads in HeLa and TAP cells.

(E–G) Diagrams showing spliced and unspliced 5′ ss intermediates associated with a constitutive exon (E), an alternative exon (F) and a U12-type intron (G).

(H) Model showing sequential co-transcriptional splicing associated with S5P CTD of Pol II.

elongating Pol II complex within a coIPed spliceosome. Our new results now establish this hypothesis (Figure 4H). Thus, both spliceosome-associated proteins (Figure 1) and snRNAs (Figure 2) selectively associate with S5P CTD Pol II. In particular the observed enrichment for U5 snRNP as well as most protein components of spliceosomal Bact and C complexes (Figures 1, 2, and S1) shows that the catalytically active spliceosome associates with S5P CTD Pol II during co-transcriptional splicing. Interestingly, we detect lower levels of U1 snRNP in our datasets. Even though U1 snRNP is a known Pol II interactor (Das et al., 2007; Harlen et al., 2016), its association is likely transient in nature, possibly because it acts as a splice site selector factor rather than playing a direct role in spliceosome-mediated catalysis (Papasaikas and Valcárcel, 2016). Furthermore, it also acts to block the recognition of cryptic polyA signals often found within large mammalian introns (Oh et al., 2017). We consistently observed that spliceosomal proteins and snRNAs were much more abundant in Pol II complexes IPed with antibodies specific for S5P CTD than antibodies specific for S2P and T4P CTD. In the case of T4P, ChIP-seq analysis suggests that this

modification is associated with transcription termination (Hintermair et al., 2012). Its lack of spliceosomal association is therefore predictable. In contrast, numerous ChIP-seq studies revealed that Pol II complexes containing S5P-modified CTD are enriched at the promoter proximal region and diminish thereafter (for a comprehensive review, see Eick and Geyer, 2013). However, a splicing-related accumulation of S5P Pol II along gene bodies was found both in HeLa cells (Batsché et al., 2006) and in yeast (Alexander et al., 2010; Chathoth et al., 2014). Moreover, a recent proteomic analysis of phospho-specific Pol II complexes in yeast revealed that the interactome of S5P-modified Pol II is enriched for spliceosomal components (Harlen et al., 2016). Our results extend these observations by showing that a S5P Pol II-associated, catalytically active spliceosome exists as a stable entity in human cells (Figure 4H). We suggest that S5P CTD is not restricted to transcription initiation but is also present during elongation and co-transcriptional splicing.

It is currently unclear which CTD kinases and phosphatases are associated with CTD phosphorylation during co-transcriptional splicing. CDK7, CDK9, and CDK12 are all known to act as CTD kinases (Bartkowiak et al., 2010; Hsin and Manley, 2012). However, these enzymes were not detected in our mNET-MS experiments. Possibly interactions between kinase and Pol II will be transient. Notably, we did detect RPAP2 phosphatase-interacting proteins RPRD1A and RPRD1B in S5P Pol II complexes (Table S1). RPRD1A and RPRD1B have been reported to act as CTD scaffolds to coordinate S5 dephosphorylation by RPAP2 (Ni et al., 2014).

Together with spliceosome components associated with Pol II complexes, our mNET-seq analysis also uncovers new information about splicing intermediates that form co-transcriptionally. We find that the vast majority of splicing events (~90%) are associated with a 5′ ss peak corresponding to the splicing intermediate formed after initial 5′ ss cleavage generated by the formation of the intron lariat (Figure 4C). Cleaved short exons (< 60 nt) can be entirely protected from MNase digestion by the spliceosome complex, allowing interrogation of whether splicing to the previously synthesized exon has occurred. Overall, we found splicing intermediates containing short exons frequently spliced to the upstream exon. As shown in Figure S4G for *OSTF1*, each of the 4 adjacent 5′ ss intermediates is associated with upstream exon spliced reads. In effect, this means that mNET-seq detects previously transcribed nascent pre-mRNA with its introns already excised but that is still in the process of active synthesis of its downstream regions. Remarkably, we observe intermediates containing the rare U12-type introns already excised (Figures 4G and S4F). Thus, although minor introns have been reported to be slowly spliced (Niemelä and Frilander, 2014), mNET-seq reveals that U12 introns are efficiently co-transcriptionally spliced. In contrast, a higher proportion of unspliced intermediates is detected in the case of alternative upstream splicing events (Figure 4F), consistent with the view that not all exon pairs in a pre-mRNA are ligated with similar kinetics (Pandya-Jones and Black, 2009; Rabani et al., 2014; Wetterberg et al., 1996) and that alternative splicing tends to be less efficient than constitutive splicing (Khodor et al., 2011, 2012).

It is notable that our observed pattern of mammalian co-transcriptional splicing differs from that proposed for *S. cerevisiae*. In yeast, splicing has been shown to occur when Pol II is still transcribing the downstream exon, implying an intron definition mechanism (Oesterreich et al., 2016). Instead, in mammalian genes, which tend to have short exons separated by very long introns, the exon definition model posits that splicing occurs after Pol II has transcribed beyond the entire exon (Keren et al., 2010). In our analysis, we show that most mammalian splicing occurs after Pol II has elongated beyond the downstream 5′ ss. Even so, we predict that splicing is a normally rapid process that occurs as soon as the elongating Pol II completes transcription of a particular exon.

STAR★METHODS

Detailed methods are provided in the online version of this paper and include the following:

- KEY RESOURCES TABLE
- CONTACT FOR REAGENT AND RESOURCE SHARING
- EXPERIMENTAL MODEL AND SUBJECT DETAILS
- METHOD DETAILS
  - Short and long RNAs mNET-seq
  - Mass spectrometry with filter-aid sample preparation
  - Glycerol gradient experiment
- QUANTIFICATION AND STATISTICAL ANALYSIS
  - mNET-seq and RNA-seq data processing
  - Nucleoplasmic RNA-seq data analysis
  - Annotation
  - mNET-Mass Spectrometry data analysis
  - snRNA read counts
  - mNET-seq peak finding analysis
  - Percent-spliced-in (PSI) calculation
  - Identification of spliced events
  - Splicing associated with 5′ ss peak
  - mNET-seq read density analysis
- DATA AND SOFTWARE AVAILABILITY

SUPPLEMENTAL INFORMATION

Supplemental Information includes four figures and one table and can be found with this article online at https://doi.org/10.1016/j.molcel.2018.09.004.

ACKNOWLEDGMENTS

We thank members of the N.J.P. and M.C.-F. groups for critical discussion. We are indebted to Grzegorz Kdula (University of Edinburgh, UK) for bioinformatics insight. This work was supported by funding to N.J.P. (Wellcome Trust Investigator Award [107928/Z/15/Z] and ERC Advanced [339270] grants) and to M.C.-F. (Fundação Ciência e Tecnologia, Portugal; grant PTDC/BEX-BCM/5899/2014).

AUTHOR CONTRIBUTIONS

T.N. performed all molecular biology experiments. K.R., aided by A.R.G. and T.G., carried out the bioinformatic analyses. T.N., N.J.P., and M.C.-F. conceived the project and wrote the paper.

DECLARATION OF INTERESTS

The authors declare no competing interests.

Received: November 16, 2017
Revised: July 23, 2018
Accepted: August 31, 2018
Published: October 18, 2018

REFERENCES

Alexander, R.D., Innocente, S.A., Barrass, J.D., and Beggs, J.D. (2010). Splicing-dependent RNA polymerase pausing in yeast. Mol. Cell *40*, 582–593.

Alioto, T.S. (2007). U12DB: a database of orthologous U12-type spliceosomal introns. Nucleic Acids Res. *35*, D110–D115.

Andrecka, J., Treutlein, B., Arcusa, M.A., Muschielok, A., Lewis, R., Cheung, A.C., Cramer, P., and Michaelis, J. (2009). Nano positioning system reveals the course of upstream and nontemplate DNA within the RNA polymerase II elongation complex. Nucleic Acids Res. *37*, 5803–5809.

Ast, G. (2004). How did alternative splicing evolve? Nat. Rev. Genet. *5*, 773–782.

Barbosa-Morais, N.L., Irimia, M., Pan, Q., Xiong, H.Y., Gueroussov, S., Lee, L.J., Slobodeniuc, V., Kutter, C., Watt, S., Colak, R., et al. (2012). The evolutionary landscape of alternative splicing in vertebrate species. Science *338*, 1587–1593.

Barrass, J.D., and Beggs, J.D. (2003). Splicing goes global. Trends Genet. *19*, 295–298.

Bartkowiak, B., Liu, P., Phatnani, H.P., Fuda, N.J., Cooper, J.J., Price, D.H., Adelman, K., Lis, J.T., and Greenleaf, A.L. (2010). CDK12 is a transcription elongation-associated CTD kinase, the metazoan ortholog of yeast Ctk1. Genes Dev. *24*, 2303–2316.

Batsché, E., Yaniv, M., and Muchardt, C. (2006). The human SWI/SNF subunit Brm is a regulator of alternative splicing. Nat. Struct. Mol. Biol. *13*, 22–29.

Chathoth, K.T., Barrass, J.D., Webb, S., and Beggs, J.D. (2014). A splicing-dependent transcriptional checkpoint associated with prespliceosome formation. Mol. Cell *53*, 779–790.

Chen, M., and Manley, J.L. (2009). Mechanisms of alternative splicing regulation: insights from molecular and genomics approaches. Nat. Rev. Mol. Cell Biol. *10*, 741–754.

Chen, W., Moore, J., Ozadam, H., Shulha, H.P., Rhind, N., Weng, Z., and Moore, M.J. (2018). Transcriptome-wide Interrogation of the Functional Intronome by Spliceosome Profiling. Cell *173*, 1031–1044.e1013.

Churchman, L.S., and Weissman, J.S. (2011). Nascent transcript sequencing visualizes transcription at nucleotide resolution. Nature *469*, 368–373.

Das, R., Yu, J., Zhang, Z., Gygi, M.P., Krainer, A.R., Gygi, S.P., and Reed, R. (2007). SR proteins function in coupling RNAP II transcription to pre-mRNA splicing. Mol. Cell *26*, 867–881.

Dujardin, G., Lafaille, C., de la Mata, M., Marasco, L.E., Muñoz, M.J., Le Jossic-Corcos, C., Corcos, L., and Kornblihtt, A.R. (2014). How slow RNA polymerase II elongation favors alternative exon skipping. Mol. Cell *54*, 683–690.

Eick, D., and Geyer, M. (2013). The RNA polymerase II carboxy-terminal domain (CTD) code. Chem. Rev. *113*, 8456–8490.

Harlen, K.M., Trotta, K.L., Smith, E.E., Mosaheb, M.M., Fuchs, S.M., and Churchman, L.S. (2016). Comprehensive RNA Polymerase II Interactomes Reveal Distinct and Varied Roles for Each Phospho-CTD Residue. Cell Rep. *15*, 2147–2158.

Hart, T., Komori, H.K., LaMere, S., Podshivalova, K., and Salomon, D.R. (2013). Finding the active genes in deep RNA-seq gene expression studies. BMC Genomics *14*, 778.

Heidemann, M., Hintermair, C., Voß, K., and Eick, D. (2013). Dynamic phosphorylation patterns of RNA polymerase II CTD during transcription. Biochim. Biophys. Acta *1829*, 55–62.

Hintermair, C., Heidemann, M., Koch, F., Descostes, N., Gut, M., Gut, I., Fenouil, R., Ferrier, P., Flatley, A., Kremmer, E., et al. (2012). Threonine-4 of mammalian RNA polymerase II CTD is targeted by Polo-like kinase 3 and required for transcriptional elongation. EMBO J. *31*, 2784–2797.

Hollander, D., Naftelberg, S., Lev-Maor, G., Kornblihtt, A.R., and Ast, G. (2016). How Are Short Exons Flanked by Long Introns Defined and Committed to Splicing? Trends Genet. *32*, 596–606.

Hsin, J.P., and Manley, J.L. (2012). The RNA polymerase II CTD coordinates transcription and RNA processing. Genes Dev. *26*, 2119–2137.

Hubner, N.C., and Mann, M. (2011). Extracting gene function from protein-protein interactions using Quantitative BAC InteraCtomics (QUBIC). Methods *53*, 453–459.

Hubner, N.C., Bird, A.W., Cox, J., Splettstoesser, B., Bandilla, P., Poser, I., Hyman, A., and Mann, M. (2010). Quantitative proteomics combined with BAC TransgeneOmics reveals in vivo protein interactions. J. Cell Biol. *189*, 739–754.

Karolchik, D., Hinrichs, A.S., Furey, T.S., Roskin, K.M., Sugnet, C.W., Haussler, D., and Kent, W.J. (2004). The UCSC Table Browser data retrieval tool. Nucleic Acids Res. *32*, D493–D496.

Keren, H., Lev-Maor, G., and Ast, G. (2010). Alternative splicing and evolution: diversification, exon definition and function. Nat. Rev. Genet. *11*, 345–355.

Kettenberger, H., Armache, K.J., and Cramer, P. (2004). Complete RNA polymerase II elongation complex structure and its interactions with NTP and TFIIS. Mol. Cell *16*, 955–965.

Khodor, Y.L., Rodriguez, J., Abruzzi, K.C., Tang, C.H., Marr, M.T., 2nd, and Rosbash, M. (2011). Nascent-seq indicates widespread cotranscriptional pre-mRNA splicing in Drosophila. Genes Dev. *25*, 2502–2512.

Khodor, Y.L., Menet, J.S., Tolan, M., and Rosbash, M. (2012). Cotranscriptional splicing efficiency differs dramatically between Drosophila and mouse. RNA *18*, 2174–2186.

Kim, D., Pertea, G., Trapnell, C., Pimentel, H., Kelley, R., and Salzberg, S.L. (2013). TopHat2: accurate alignment of transcriptomes in the presence of insertions, deletions and gene fusions. Genome Biol. *14*, R36.

Lund, E., and Dahlberg, J.E. (1992). Cyclic 2′ ,3′ -phosphates and nontemplated nucleotides at the 3′ end of spliceosomal U6 small nuclear RNA's. Science *255*, 327–330.

Martin, M. (2011). Cutadapt removes adapter sequences from high-throughput sequencing reads. EMBnet.journal. https://doi.org/10.14806/ej.17.1.200.

Martinez-Rucobo, F.W., Kohler, R., van de Waterbeemd, M., Heck, A.J., Hemann, M., Herzog, F., Stark, H., and Cramer, P. (2015). Molecular Basis of Transcription-Coupled Pre-mRNA Capping. Mol. Cell *58*, 1079–1089.

Mayer, A., Heidemann, M., Lidschreiber, M., Schreieck, A., Sun, M., Hintermair, C., Kremmer, E., Eick, D., and Cramer, P. (2012). CTD tyrosine phosphorylation impairs termination factor recruitment to RNA polymerase II. Science *336*, 1723–1725.

Muñoz, M.J., de la Mata, M., and Kornblihtt, A.R. (2010). The carboxy terminal domain of RNA polymerase II and alternative splicing. Trends Biochem. Sci. *35*, 497–504.

Ni, Z., Xu, C., Guo, X., Hunter, G.O., Kuznetsova, O.V., Tempel, W., Marcon, E., Zhong, G., Guo, H., Kuo, W.W., et al. (2014). RPRD1A and RPRD1B are human RNA polymerase II C-terminal domain scaffolds for Ser5 dephosphorylation. Nat. Struct. Mol. Biol. *21*, 686–695.

Niemelä, E.H., and Frilander, M.J. (2014). Regulation of gene expression through inefficient splicing of U12-type introns. RNA Biol. *11*, 1325–1329.

Nieto Moreno, N., Giono, L.E., Cambindo Botto, A.E., Muñoz, M.J., and Kornblihtt, A.R. (2015). Chromatin, DNA structure and alternative splicing. FEBS Lett. *589*, 3370–3378.

Nojima, T., Gomes, T., Grosso, A.R.F., Kimura, H., Dye, M.J., Dhir, S., Carmo-Fonseca, M., and Proudfoot, N.J. (2015). Mammalian NET-Seq Reveals Genome-wide Nascent Transcription Coupled to RNA Processing. Cell *161*, 526–540.

Nojima, T., Gomes, T., Carmo-Fonseca, M., and Proudfoot, N.J. (2016). Mammalian NET-seq analysis defines nascent RNA profiles and associated RNA processing genome-wide. Nat. Protoc. *11*, 413–428.

Oesterreich, F.C., Herzel, L., Straube, K., Hujer, K., Howard, J., and Neugebauer, K.M. (2016). Splicing of Nascent RNA Coincides with Intron Exit from RNA Polymerase II. Cell *165*, 372–381.

Oh, J.M., Di, C., Venters, C.C., Guo, J., Arai, C., So, B.R., Pinto, A.M., Zhang, Z., Wan, L., Younis, I., and Dreyfuss, G. (2017). U1 snRNP telescripting regulates a size-function-stratified human genome. Nat. Struct. Mol. Biol. *24*, 993–999.

Pandya-Jones, A., and Black, D.L. (2009). Co-transcriptional splicing of constitutive and alternative exons. RNA *15*, 1896–1908.

Papasaikas, P., and Valcárcel, J. (2016). The Spliceosome: The Ultimate RNA Chaperone and Sculptor. Trends Biochem. Sci. *41*, 33–45.

Patel, A.A., and Steitz, J.A. (2003). Splicing double: insights from the second spliceosome. Nat. Rev. Mol. Cell Biol. *4*, 960–970.

Quinlan, A.R., and Hall, I.M. (2010). BEDTools: a flexible suite of utilities for comparing genomic features. Bioinformatics *26*, 841–842.

Rabani, M., Raychowdhury, R., Jovanovic, M., Rooney, M., Stumpo, D.J., Pauli, A., Hacohen, N., Schier, A.F., Blackshear, P.J., Friedman, N., et al. (2014). High-resolution sequencing and modeling identifies distinct dynamic RNA regulatory strategies. Cell *159*, 1698–1710.

Rosenbloom, K.R., Armstrong, J., Barber, G.P., Casper, J., Clawson, H., Diekhans, M., Dreszer, T.R., Fujita, P.A., Guruvadoo, L., Haeussler, M., et al. (2015). The UCSC Genome Browser database: 2015 update. Nucleic Acids Res. *43*, D670–D681.

Schlackow, M., Nojima, T., Gomes, T., Dhir, A., Carmo-Fonseca, M., and Proudfoot, N.J. (2017). Distinctive Patterns of Transcription and RNA Processing for Human lincRNAs. Mol. Cell *65*, 25–38.

Schwartz, S., and Ast, G. (2010). Chromatin density and splicing destiny: on the cross-talk between chromatin structure and splicing. EMBO J. *29*, 1629–1636.

Trapnell, C., Williams, B.A., Pertea, G., Mortazavi, A., Kwan, G., van Baren, M.J., Salzberg, S.L., Wold, B.J., and Pachter, L. (2010). Transcript assembly and quantification by RNA-Seq reveals unannotated transcripts and isoform switching during cell differentiation. Nat. Biotechnol. *28*, 511–515.

Turunen, J.J., Niemelä, E.H., Verma, B., and Frilander, M.J. (2013). The significant other: splicing by the minor spliceosome. Wiley Interdiscip. Rev. RNA *4*, 61–76.

Wallace, E.W.J., and Beggs, J.D. (2017). Extremely fast and incredibly close: cotranscriptional splicing in budding yeast. RNA *23*, 601–610.

Wetterberg, I., Baurén, G., and Wieslander, L. (1996). The intranuclear site of excision of each intron in Balbiani ring 3 pre-mRNA is influenced by the time remaining to transcription termination and different excision efficiencies for the various introns. RNA *2*, 641–651.

Zaborowska, J., Egloff, S., and Murphy, S. (2016). The pol II CTD: new twists in the tail. Nat. Struct. Mol. Biol. *23*, 771–777.

STAR★METHODS

KEY RESOURCES TABLE

REAGENT OR RESOURCE	SOURCE	IDENTIFIER
Antibodies		
Mouse monoclonal anti-Pol II CTD, Total	MBL international	Cat# MABI0601; RRID: AB_2728735
Mouse monoclonal anti-Pol II CTD, phospho Ser2	MBL international	Cat# MABI0602; RRID: AB_2747403
Mouse monoclonal anti-Pol II CTD, phospho Ser5	MBL international	Cat# MABI0603; RRID: AB_2728736
Rabbit polyclonal anti-U1A	Abcam	Cat# ab155054; RRID: AB_2747404
Rabbit polyclonal anti-U1C	Bethyl	Cat# A303-947A; RRID: AB_2620296
Rabbit polyclonal anti-SNRNP200	Bethyl	Cat# A303-454A-T; RRID: AB_10953802
Rabbit polyclonal anti-SNRNP116	Bethyl	Cat# 300-957A; RRID: AB_805780
Rabbit polyclonal anti-Spt6	Novus Biologicals	Cat#NB100-2582; RRID: AB_2196402
Rabbit polyclonal anti-eIF4A3	Bethyl	Cat# 302-981A-T; RRID: AB_10748369
Rabbit polyclonal anti-SF3B3	Bethyl	Cat# 302-508A; RRID: AB_1966103
Mouse monoclonal anti-H3	MBL international	Cat# MABI0301; RRID: AB_11142498
Mouse monoclonal anti-trymethyl H3 (Lys36)	MBL international	Cat# MABI0333; RRID: AB_11126731
Mouse monoclonal anti-trymethyl H3 (Lys9)	GeneTex	Cat# MABI0308 (GTX50900); RRID: AB_11142491
Deposited Data		
Raw sequencing data	This paper	GEO: GSE106881
Raw mass spectrometry data	This paper	ProteomeXchange: PXD008197
Raw deposited images	Mendeley	https://doi.org/10.17632/cy8mj7sxp9.1
Re-analyzed mNET-seq data	(Nojima et al., 2015; Schlackow et al., 2017)	GEO: GSE60358 and GSE81662
Re-analyzed ChrRNA-seq and NpRNA-seq data	(Nojima et al., 2015; Schlackow et al., 2017)	GEO: GSE60358 and GSE81662
Experimental Models: Cell Lines		
HeLa (human)	Proudfoot Lab	N/A
TAP (mouse)	Carmo-Fonseca Lab	N/A
Other		
TruSeq small RNA library prep kit	Illumina	Cat# 15016911
NEBNext small RNA library prep kit for Illumina	NEB	Cat# E7300S
Software and Algorithms		
Cutadapt(v1.8.3)	(Martin, 2011)	http://cutadapt.readthedocs.io/en/stable/installation.html
Tophat(v2.0.13)	(Kim et al., 2013; Trapnell et al., 2010)	http://ccb.jhu.edu/software/tophat/index.shtml
Cufflinks(v2.2.0)	(Trapnell et al., 2010)	http://cole-trapnell-lab.github.io/cufflinks/getting_started/
bedtools (v2.25.0)	(Quinlan and Hall, 2010)	http://bedtools.readthedocs.io/en/latest/content/installation.html

CONTACT FOR REAGENT AND RESOURCE SHARING

Further information and requests for resources and reagents should be directed to the lead contact, Nicholas Proudfoot (nicholas.proudfoot@path.ox.ac.uk).

EXPERIMENTAL MODEL AND SUBJECT DETAILS

HeLa cells and TAP cells were maintained in Dulbecco's Modified Eagle's Medium (DMEM) and RPMI, respectively, with 10% fetal bovine serum (FBS). Empigen treatment and *in vivo* splicing inhibition with Pla-B were as previously described (Nojima et al., 2015; Schlackow et al., 2017).

METHOD DETAILS

Short and long RNAs mNET-seq

Four 15 cm plates of HeLa cells (3.2x10^7 cells) were used for mNET-seq of fractionated RNAs. Immunoprecipitated samples with Pol II antibodies (MABI0602 and MABI0603) from solubilized chromatin of HeLa cells were washed eight times with 1 mL of NET-2 buffer (50 mM Tris-HCl pH7.4, 150 mM NaCl and 0.05% NP-40). 5′ phosphate is added to IPed RNAs using T4 PNK for 6 min as described before (Nojima et al., 2016). The 5′ p-RNAs were purified according to a protocol of TRI reagent (Sigma-Aldrich). The purified RNAs were run on denaturing gel (6% Novex TBE-Urea gel, Thermo-Fisher Scientific) with 1xTBE buffer (Thermo-Fisher Scientific) at 300 mA for 10 min. The two RNA fractions (20-60nt and 60-160nt) were size-selected on the gel. The RNAs were put into a 0.5mL tube with three small holes that were made by 26G needle and the 0.5mL tube was put onto a 1.5mL tube. The gels were slushed by centrifuge at 16,000 g for 1 min. The slushed RNAs were incubated with 400 μL RNA elution buffer (1M NaOAc and 1mM EDTA) at 25°C for 1 hour. The solution with gel was put into Spin X column (Coaster #8160) with 2 glass filters (Whatman #1823-010) and the column was centrifuged at 16,000 g for 1 min. The flow through (400 μL) was purified with ethanol precipitation. Finally, RNA libraries were prepared using Illumina TruSeq small RNA library prep kit (Illumina) or NEBNext small RNA library prep kit for Illumina (NEB) with minimum cycles of PCR (12~15 cycles). The expected sizes of PCR products from short and long RNAs are 150-180bp and 180-280bp, respectively. The libraries were sequenced using Hiseq4000 (Illumina) at Wellcome Trust Centre of Human Genetics, Oxford.

Mass spectrometry with filter-aid sample preparation

Four 15 cm plates of HeLa cells (3.2x10^7 cells) were used for mass spectrometry experiment. Immunoprecipitated samples with Pol II antibodies from solubilized chromatin of HeLa cells were washed eight times with 1 mL of NET-2 buffer (50 mM Tris-HCl pH7.4, 150 mM NaCl and 0.05% NP-40) and then once with 500 μL of NET buffer (50 mM Tris-HCl pH7.4 and 150 mM NaCl). After NET buffer was removed completely, washed beads were incubated in 300 μL of lysis buffer (8 M Urea and 100 mM Triethylammonium bicarbonate buffer (TEAB) pH 8.0) for 30 min at room temperature. The mixture with beads was transferred to a Vivacon 10 kDa MWCO centrifugal concentrator (viva) and centrifuged at 16,000 g for 15 min at room temperature. The filter on tube was washed with 200 μL of lysis buffer twice by centrifugation at 16,000 g for 15 min at room temperature. The samples were incubated in 200 μL of reduction buffer (8 M Urea, 100 mM TEAB pH 8.0 and 20 mM Tris (2-carboxyethyl)phosphine hydrochloride solution pH 7.0) on the filter for 30 min, followed by centrifugation at 16,000 g for 15 min at room temperature. Next, 30 min incubation with 200 μL of alkylation buffer (8 M Urea, 100 mM TEAB pH 8.0 and 20 mM chloroiodoacetamide) was performed in the dark place. The reaction solution was removed by centrifugation at 16,000 g for 15 min at room temperature. The samples were washed with wash buffer (1 M Urea, 50 mM TEAB pH 8.0) twice and then digested with 1 μg trypsin in 100 μL wash buffer for 30 min at 37°C on the filter. The trypsinization was repeated once more for over-night at 37°C. The samples were collected by 16,000 g for 15 min at room temperature as the follow through and desalted with a C18 column. Finally, the purified peptides were analyzed by electrospray tandem mass spectrometry at the central proteomics facility in University of Oxford.

Glycerol gradient experiment

6 mL of 30% glycerol solution (20 mM HEPES-KOH pH7.9, 150 mM KCl, 1.5 mM $MgCl_2$, 0.2 mM EDTA and 30% Glycerol) was overlaid with 6 mL of 10% glycerol solution (20 mM HEPES-KOH pH7.9, 150 mM KCl, 1.5 mM $MgCl_2$, 0.2 mM EDTA and 10% Glycerol). To make sequential glycerol layers, the tube was laid on the bench for 3 hours at room temperature and put the tube vertical for 1 hour in cold room. Approximately 1 mL of soluble chromatin fraction prepared as previously described was gently overlaid onto sequential glycerol solution and immediately centrifuge at 30,000 rpm for 18~20 hours at 4°C. After the ultracentrifuge, 22 fractions (500 μL each) were separated for the top of the tube. Fractions #1(top)-6, #7-14, #15-22 were pooled for 3 rough fractions called light, middle and heavy, respectively. The rest (~1 mL) of sample called fraction #23 at bottom was not used for further Pol II IP step since aggregates were observed. After Pol II IP, standard mNET-seq protocol was followed as previously described (Nojima et al., 2016).

QUANTIFICATION AND STATISTICAL ANALYSIS

mNET-seq and RNA-seq data processing

Data pre-processing was performed as previously described (Nojima et al., 2016). Read Coverage, gene elements comparison and manipulation were performed using bedtools (v2.25.0) (Quinlan and Hall, 2010). Meta-profiles, splice junction average profiles and individual profiles were generated as described (Nojima et al., 2015).

Nucleoplasmic RNA-seq data analysis

Strand-specific nucleoplasmic RNA-seq data was analyzed with Tophat(v2.0.13)/Cufflinks(v2.2.0) (Trapnell et al., 2010) using Ensembl *Homo sapiens* GRCh37.74 release as template for quantification. zFPKM values for each transcript were converted to log2 and their distribution plotted. The threshold value chosen to identify expressed genes was the local minimum between the peak of high-expressed genes and low-expressed genes as described in (Hart et al., 2013).

Annotation

Annotations for TSS, introns, exons and alternative splicing events were retrieved from Ensembl for hg19 and mm10 genome version (Karolchik et al., 2004; Rosenbloom et al., 2015). Constitutive events were retrieved from Ensembl and U12-type spliceosomal introns retrieved from U12DB through a liftOver tool to bring all genetical information to the same reference build (Alioto, 2007).

mNET-Mass Spectrometry data analysis

Spectral counts for each specific IP along with the respective mock control were used for analysis. Missing values were imputed, and data were normalized by median subtraction as described (Harlen et al., 2016). Enriched proteins were defined using a false discovery rate (FDR) of 0.05.

snRNA read counts

A list of all ENSEMBL genes was filtered to select all genes containing U1, U2, U3, U4, U5, U6 or U7 in the respective gene symbol, grouping the genes by each snRNA category. The obtained list was then manually curated to remove incorrectly classified genes. Afterward, the number of nucleoplasm-fraction RNA reads was counted for non-overlapping snRNA genes, and the ones with a log2(RPM) equal or greater than 1 for this sample were considered expressed, constituting the gene set used in the analysis. For this final snRNA set, reads were counted and normalized to RPM for every tested sample, adding 5 bases to the annotated coordinates to account for possible annotation uncertainties, and RPM values for genes belonging to the same snRNA category were added.

mNET-seq peak finding analysis

To identify spikes in the density of 3′ ends of nascent transcripts we used an algorithm that finds nucleotides where the read density is at least three standard deviations above the mean in a local region (Churchman and Weissman, 2011). Only positions with coverage of 4 or more reads were considered. To identify peaks at the 3′ end of exons and introns indicative of splicing intermediates, the number of reads at these positions was compared to the mean read density across the corresponding exon or intron. To identify peaks corresponding to Pol II pause positions along exons and introns avoiding contamination by 5′ ss splicing intermediates, we removed reads aligning to the last 3 nucleotides of exons and the first 3 nucleotides of introns. The number of reads at each nucleotide position along the exon was then compared to the mean read density across the entire exon. Peaks were identified in the annotated exons and introns of expressed genes. Exons that intersected other isoform exons were discarded and the same principle was applied for introns.

Percent-spliced-in (PSI) calculation

To obtain a measure of splicing completeness for any given exon, the percent-spliced-in (PSI) metric (Barbosa-Morais et al., 2012) was applied to nucleoplasmic RNA-seq datasets. When calculating this index, exons that have alternative 3′ or 5′ splice sites were excluded from the analysis, as well as exons overlapping other exons on the same strand and exons with less than 100bp, or which the preceding intron had less than 100bp. Finally, only junctions that had a total of at least 5 reads in both regions were included in the analysis.

Identification of spliced events

Detection of spliced events was based on spliced reads obtained in mNET-seq/S5P experiments. Reads that aligned uniquely and with no mismatch across two exons were classified as spliced reads. To reduce the possibility of fortuitous alignments, only reads that map to more than 8 nucleotides across any junction were considered. A splicing event was considered if the 3′ SS was covered by 3 or more reads.

Splicing associated with 5′ ss peak

Merely spliced or unspliced mNET-seq/S5P reads that specifically align to the last nucleotide of exons were taken into consideration. Calculation of splicing percentage was performed, on exons with a total between spliced and unspliced reads equal or above 10, in the following manner:

$$spliced\% = \frac{SR}{SR + UR} x100$$

“SR” stands for spliced reads and “UR” unspliced reads. Significance of the differences between the mean and distribution of the percentage of splicing for skipping and constitutive exons was calculated through a Wilcoxon signed-rank test and Kolmogorov-Smirnov tests, respectively.

mNET-seq read density analysis

For each expressed exon and intron, that do not intersect with another exon or intron isoform respectively, read average density was calculated as follows:

$$RAD = \frac{TR - 3nt}{EL - 3nt}$$

"TR" stands for the total number of reads along the exon and "EL" to the exon length. The last 3 nucleotides of exons and the first 3 nucleotides of introns are removed because of the high read densities associated with 5′ ss splicing intermediates. For each group of exons considered, exons without reads were not considered and the signal originating from spliced reads was removed.

DATA AND SOFTWARE AVAILABILITY

All the sequencing data and mass spectrometry data used in this work is submitted at GEO: GSE106881 and ProteomeXchange: PXD008197. Original images of western blot are available at Mendeley data https://doi.org/10.17632/cy8mj7sxp9.1. Software's used in this work are all publicly available, with the links to them in the above tables.

Molecular Cell, Volume *72*

Supplemental Information

RNA Polymerase II Phosphorylated on CTD Serine 5 Interacts with the Spliceosome during Co-transcriptional Splicing

Takayuki Nojima, Kenny Rebelo, Tomás Gomes, Ana Rita Grosso, Nicholas J. Proudfoot, and Maria Carmo-Fonseca

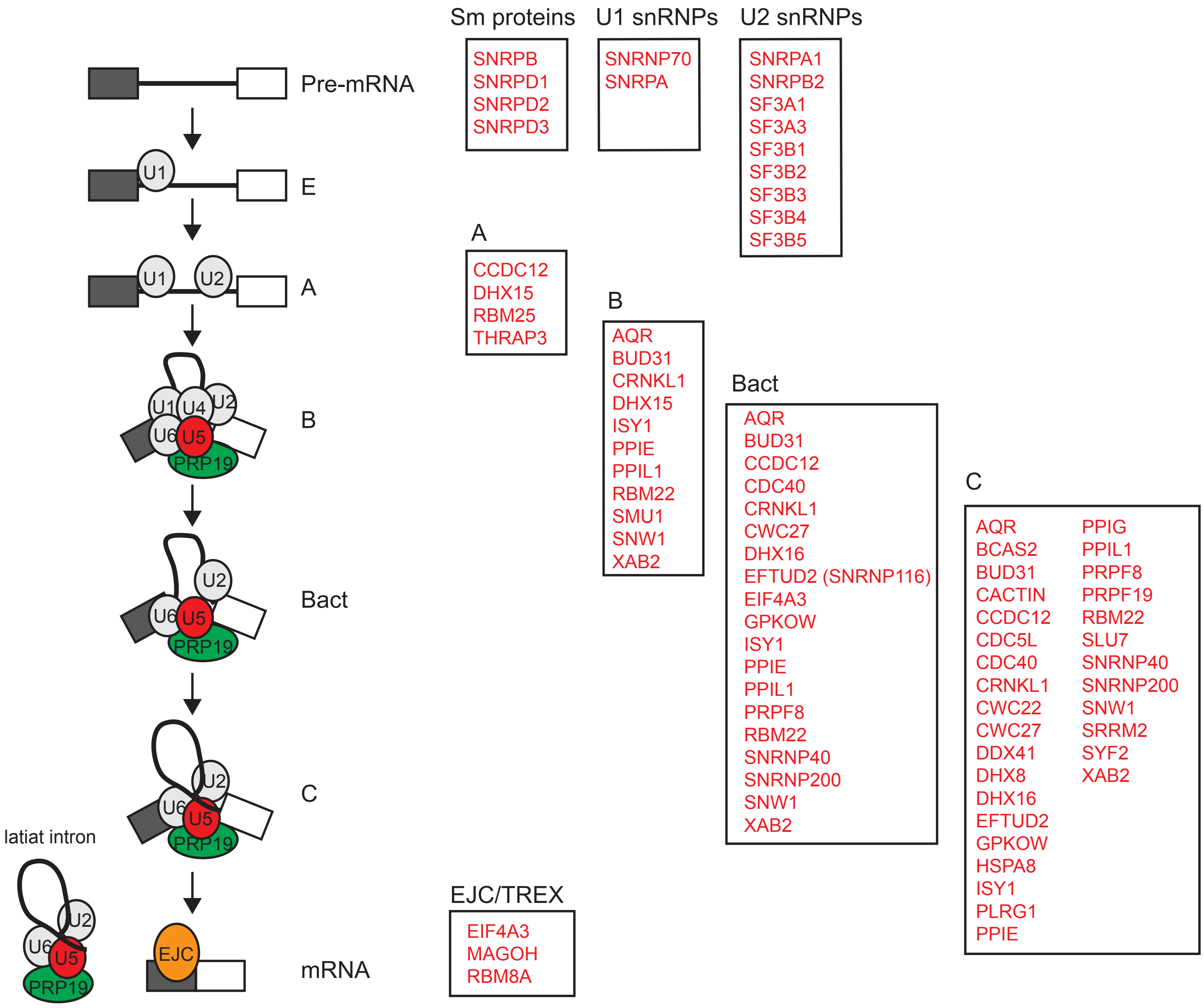

Figure S1. Spliceosome proteins associate with S5P CTD Pol II (related to Figure 1).
Schematic diagram of splicing steps with indicated U snRNAs. Peptides enriched by S5P CTD Pol II IP relative to mock IP include U snRNP proteins and components of spliceosome A, B, Bact, and C complexes.

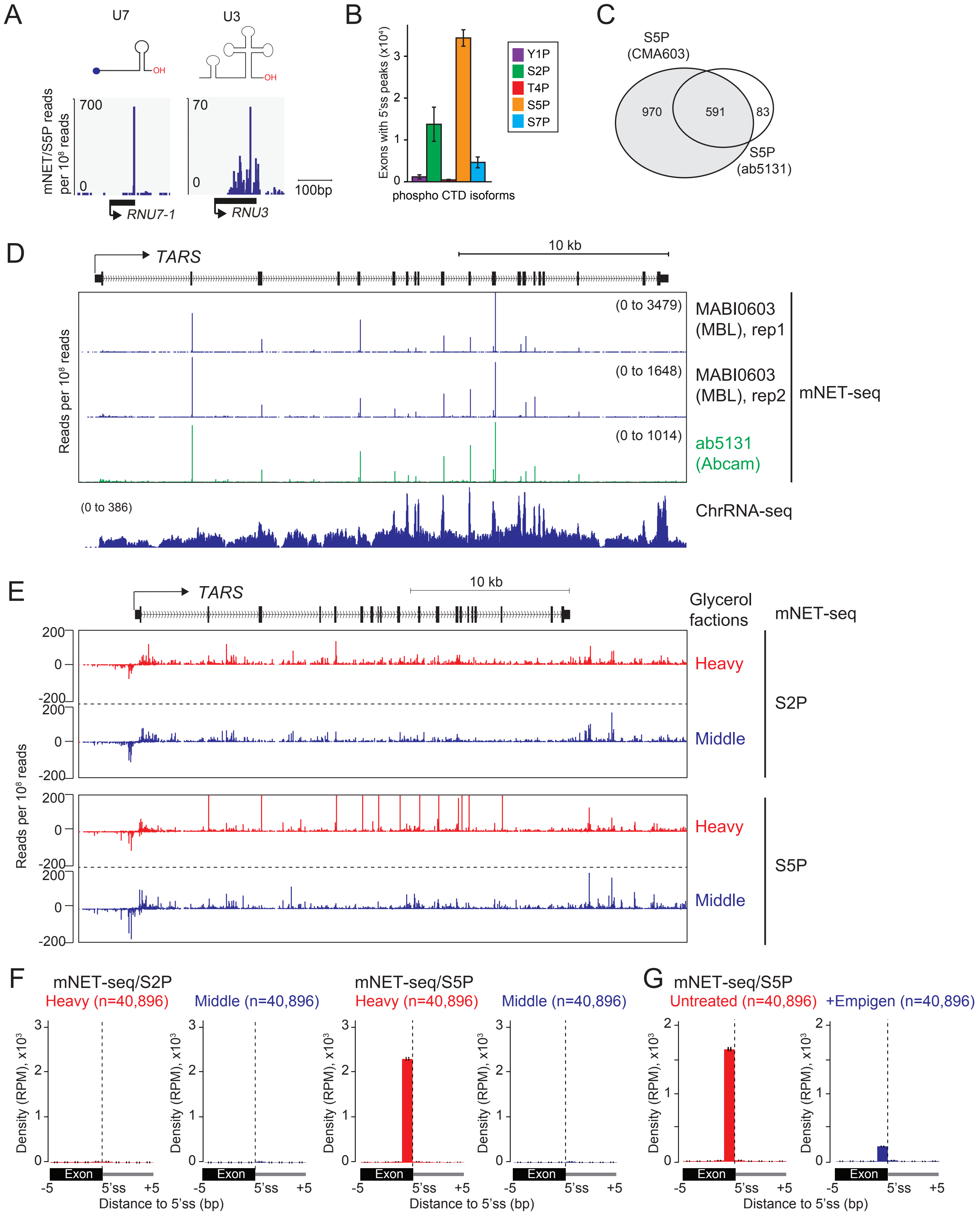

Figure S2. The catalytically active spliceosome associates with S5P CTD Pol II (related to Figures 1 and 2).
A) Enlarged mNET-seq profiles over the U7 and U3 snRNA genes using S5P CTDantibody.
B) Number of exons with a 5'ss peak detected by antibodies specific for the indicated CTD modifications (for each antibody, data was obtained from three independent libraries prepared from short RNA fragments).
C) Comparison (Venn diagram) of exons with a 5'ss peak detected using two different S5P CTD specific antibodies; MABI0603 (MBL international) and ab5131 (Abcam)
D) Comparison of mNET-seq/S5P profile over TARS using either MABI0603 (MBL international) or ab5131 (Abcam) antibodies.
E) mNET-seq/S5P and S2P profiles over TARS from heavy and middle glycerol gradient fractions.
F) Metagene analyses of mNET-seq/S5P and S2P profiles at the 5'ss of spliced events (identified in nucleoplasmic RNA-seq datasets) from heavy and middle glycerol gradient fractions.
G) Metagene analyses of mNET-seq/S5P profiles at the 5'ss of spliced events (identified innucleoplasmic RNA-seq datasets) from untreated and empigen-treated samples.

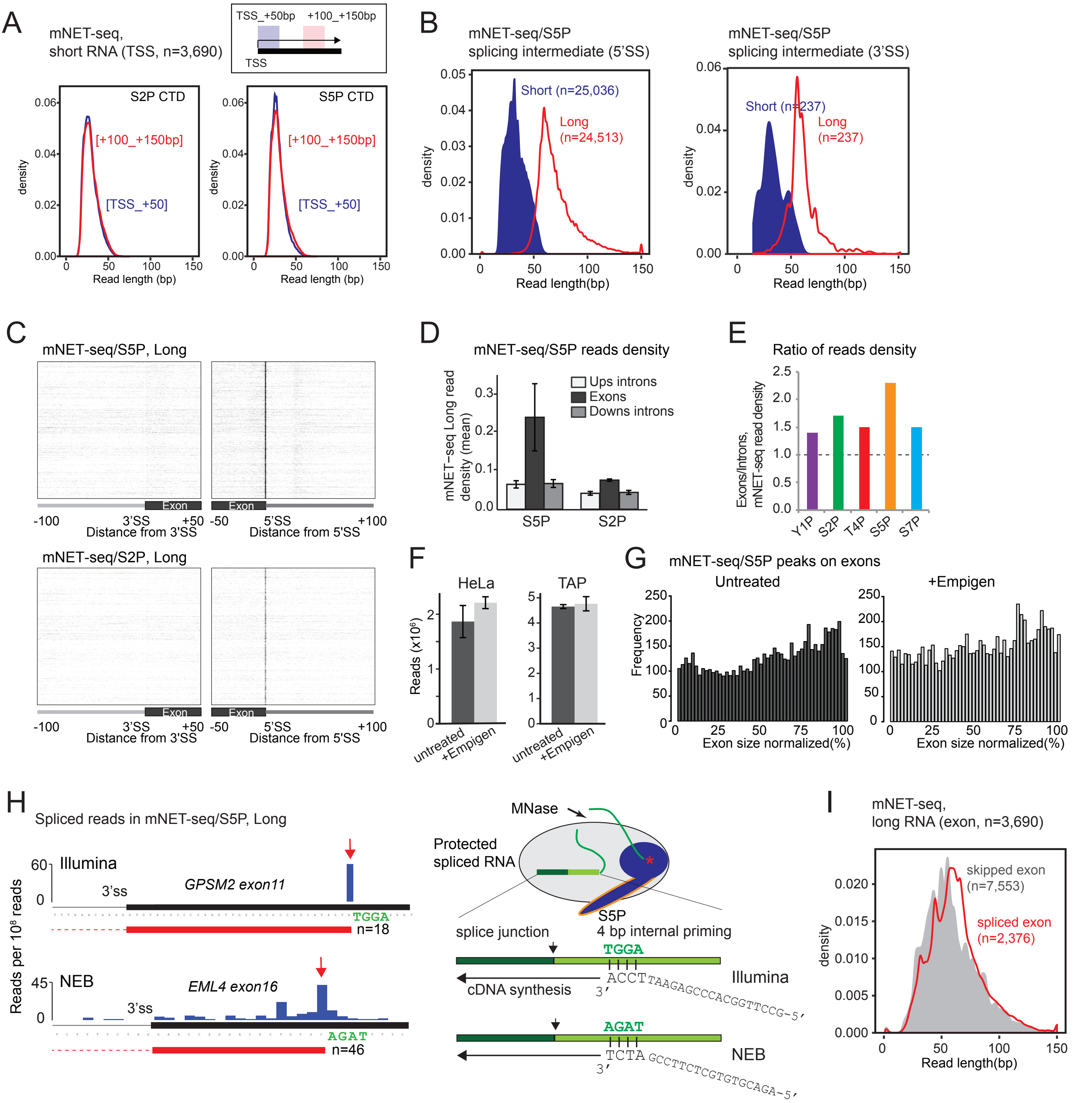

Figure S3. Elongating RNA Pol II footprints (related to Figure 3).
A) Length of mNET-seq/S5P and S2P reads mapping within either the first 50 nts or between nts 100 and 150 for 3,690 exons with length >200 nts (data from three independent libraries prepared from short RNA fragments).
B) Length of reads corresponding to 5'ss and 3'ss splicing intermediates (data from three independent libraries prepared from short and long RNA fragments).
C) Heatmaps of mNET-seq/S5P and S2P long RNA reads around 3’ss and 5’ss
D) Quantification of density of mNET-seq/S5P and S2P Long RNA reads on upstream (Ups) intron, exon and downstream (Downs) intron.
E) Ratio of mNET-seq density on exons and introns on all CTD phosphorylation isoforms
F) Quantification of mNET-seq/S5P reads of HeLa and TAP cells on exons with or without empigen treatment.
G) Frequency of mNET-seq/S5P peaks on normalized HeLa exons with or without empigen treatment.
H) Representative examples of 3’ ends of spliced reads from libraries prepared with Illumina or NEB cDNA primers. Red arrow denotes position where most 3' ends map. Specific sequence present downstream of this position (as indicated) corresponds to the 3' ends of either Illumina or NEB cDNA primers. Diagram (on the right) illustrating internal priming of spliced transcripts associated with S5P CTD Pol II.
I) Length of reads mapping within included and skipped exons (data from three independent mNET-seq/S5P libraries prepared from long RNA fragments). Reads mapping to the last 3 nts of each exon were excluded to avoid contamination with 5'ss splicing intermediates.

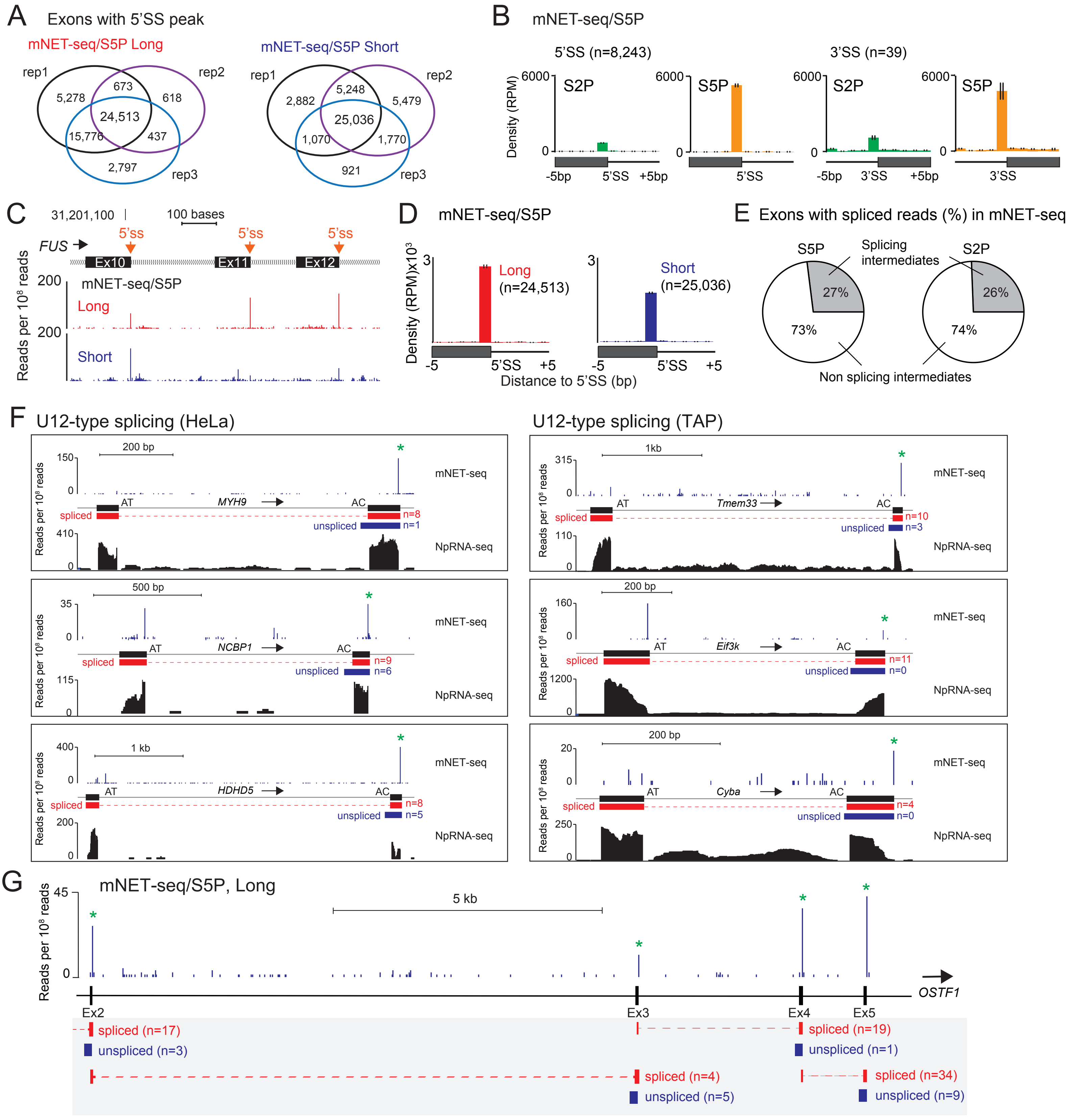

Figure S4. Sequential co-transcriptional splicing (related to Figure 4).

A) Venn diagrams showing exons with 5'ss peaks in three independent mNET-seq/S5P libraries prepared from long and short RNA fragments.

B) Metagene analysis of 5'ss and 3'ss peaks detected simultaneously in mNET-seq/S5P and mNETseq/S2P libraries.

C) mNET-seq/S5P profiles over the exon 10-12 region of FUS. Comparison of profiles obtained in libraries prepared with long and short RNA fragments.

D) Metagene analysis of 5'ss peaks detected in mNET-seq/S5P libraries prepared from long and short RNA fragments.

E) Pies depict the proportion of spliced exons associated with 5'ss splicing intermediates (data from three independent mNET-seq/S5P and S2P libraries prepared from long RNA fragments). Only events covered by a minimum of 3 spliced reads were considered.

F) Examples of 5'ss splicing intermediates with U12-type splicing in HeLa (left) and TAP cells (right). Data from three mNET-seq/S5P libraries prepared from long RNA fragments.

G) OSTF1 as an example of sequential splicing. Number of spliced and unspliced reads mapping to the 5'ss downstream of the indicated exons is shown.
